# Dimerization-Induced Allosteric Changes of the Oxyanion-Hole Loop Activate the Pseudorabies Virus Assemblin pUL26N, a Herpesvirus Serine Protease

**DOI:** 10.1371/journal.ppat.1005045

**Published:** 2015-07-10

**Authors:** Martin Zühlsdorf, Sebastiaan Werten, Barbara G. Klupp, Gottfried J. Palm, Thomas C. Mettenleiter, Winfried Hinrichs

**Affiliations:** 1 Institute of Biochemistry, University of Greifswald, Greifswald, Germany; 2 Institute of Molecular Virology and Cell Biology, Friedrich-Loeffler-Institut, Greifswald—Insel Riems, Germany; University of California at Los Angeles, UNITED STATES

## Abstract

Herpesviruses encode a characteristic serine protease with a unique fold and an active site that comprises the unusual triad Ser-His-His. The protease is essential for viral replication and as such constitutes a promising drug target. In solution, a dynamic equilibrium exists between an inactive monomeric and an active dimeric form of the enzyme, which is believed to play a key regulatory role in the orchestration of proteolysis and capsid assembly. Currently available crystal structures of herpesvirus proteases correspond either to the dimeric state or to complexes with peptide mimetics that alter the dimerization interface. In contrast, the structure of the native monomeric state has remained elusive. Here, we present the three-dimensional structures of native monomeric, active dimeric, and diisopropyl fluorophosphate-inhibited dimeric protease derived from pseudorabies virus, an alphaherpesvirus of swine. These structures, solved by X-ray crystallography to respective resolutions of 2.05, 2.10 and 2.03 Å, allow a direct comparison of the main conformational states of the protease. In the dimeric form, a functional oxyanion hole is formed by a loop of 10 amino-acid residues encompassing two consecutive arginine residues (Arg136 and Arg137); both are strictly conserved throughout the herpesviruses. In the monomeric form, the top of the loop is shifted by approximately 11 Å, resulting in a complete disruption of the oxyanion hole and loss of activity. The dimerization-induced allosteric changes described here form the physical basis for the concentration-dependent activation of the protease, which is essential for proper virus replication. Small-angle X-ray scattering experiments confirmed a concentration-dependent equilibrium of monomeric and dimeric protease in solution.

## Author Summary

Herpesviruses encode a unique serine protease, which is essential for herpesvirus capsid maturation and is therefore an interesting target for drug development. In solution, this protease exists in an equilibrium of an inactive monomeric and an active dimeric form. All currently available crystal structures of herpesvirus proteases represent complexes, particularly dimers. Here we show the first three-dimensional structure of the native monomeric form in addition to the native and the chemically inactivated dimeric form of the protease derived from the porcine herpesvirus pseudorabies virus. Comparison of the monomeric and dimeric form allows predictions on the structural changes that occur during dimerization and shed light onto the process of protease activation. These new crystal structures provide a rational base to develop drugs preventing dimerization and therefore impeding herpesvirus capsid maturation. Furthermore, it is likely that this mechanism is conserved throughout the herpesviruses.

## Introduction

The family *Herpesviridae* is divided into the three subfamilies alpha-, beta- and gammaherpesviruses. These infectious agents cause a variety of diseases in many different hosts including humans. Pseudorabies virus (PrV) is a neurotropic porcine alphaherpesvirus [1] and the causative agent of Aujeszky's disease. The pig is the only susceptible species that can survive a PrV infection depending on the age of the animal and virulence of the virus, while most other mammals die within a few days. Only higher primates including humans and equids are resistant to infection. Due to its broad host range PrV has become an important model system to study herpesvirus biology in cell culture and in the natural host. PrV genome organization and protein content exhibit significant homology to that of the human herpes simplex virus type 1 (HSV-1) [2,3], which is among the best-studied herpesviruses.

**Fig 1:**
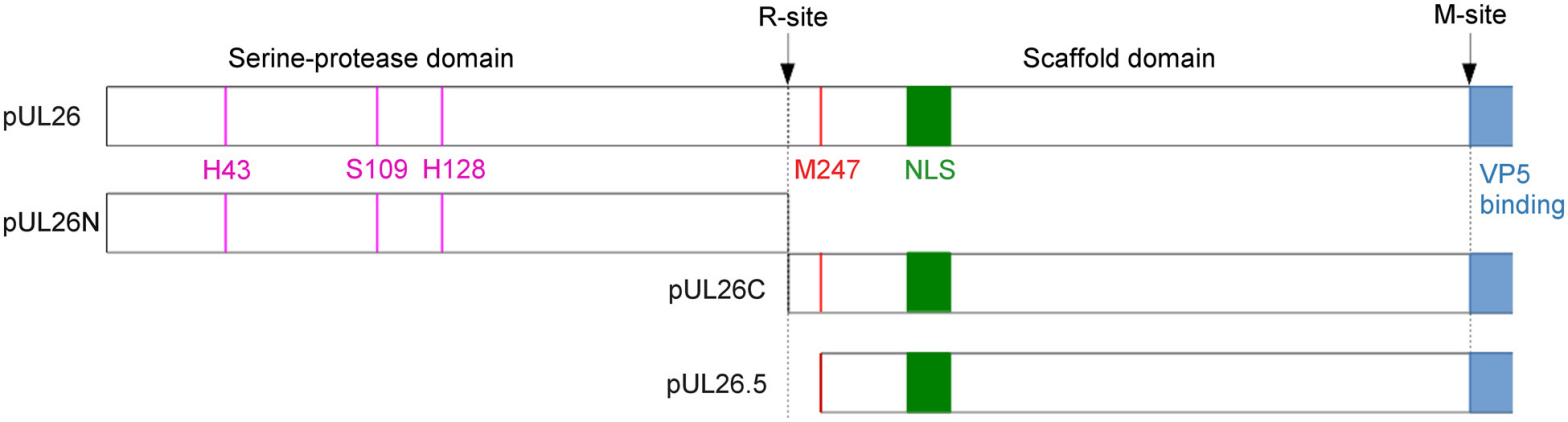
Cleavage sites and products of PrV pUL26 and pUL26.5. Schematic overview of the PrV UL26/26.5 gene products and their processing. Catalytic residues are shown in pink, the position of the nuclear localization sequence (NLS) is shown in green, the predicted major capsid protein (VP5) binding-region is shown in blue and the start of pUL26.5 is shown in red. Cleavage of PrV pUL26 at the R-site releases protease pUL26N from the scaffolding protein pUL26C, and cleavage of pUL26C/pUL26.5 at the putative M-site removes C-terminally 37 amino-acid residues thereby freeing them from interaction with the major capsid protein. The PrV R- and M-sites are most likely YLQA-T(226) and presumably VQA-S(488), respectively, as predicted from sequence alignments with the protease homologs of other herpesviruses [20,21].

Capsid assembly of HSV-1 has been intensively analyzed. However, since herpesvirus capsid proteins are well conserved, it is very likely that the process leading to mature, DNA-filled nucleocapsids is also similar. The proteolytic activity of the serine protease is essential for this process [4]. In HSV-1 and PrV, this protease is encoded by the UL26 gene [5], which is the longest open reading frame in a family of in-frame overlapping genes [5–8]. UL26 overlaps in frame with UL26.5 [3]. UL26 and UL26.5 possess identical 3'-termini, which encode a scaffold protein while the unique 5'-terminus of UL26 contains the protease domain. There are at least two target sites for the protease in the full-length UL26 protein (pUL26) [8,9]. Autoproteolytic activity at the release site (R-site) results in release of the N-terminal protease domain (pUL26N, also called VP24 or generic: assemblin) and the C-terminal part containing the scaffold protein (pUL26C, also called VP21 or generic: assembly protein) [10]. Due to the presence of a linker region pUL26C is 21 amino-acid residues longer than pUL26.5 (Fig 1). Near the C-terminus of the scaffold protein is the maturational site (M-site) where pUL26.5 and pUL26C are cleaved [10]. The scaffold protein binds to the major capsid protein pUL19 (VP5) and directs it to the nucleus [11–17]. During capsid assembly, the scaffold protein forms a scaffold core with the major capsid protein bound to the C-termini of pUL26/pUL26C/pUL26.5 [17]. When the capsid is fully assembled the scaffold is cleaved at its M-site releasing the ring-like scaffold structure from the capsid, which is then expelled during DNA packaging. In contrast, the protease remains in the nucleocapsid [18]. Without protease activity, the scaffold remains in the capsid resulting in capsids without viral DNA designated as B-capsids. Upon activation of the protease, the B-capsids mature and subsequent steps of viral replication occur as shown with a temperature-sensitive HSV-1 pUL26 mutant [19].

The function of pUL26.5 can be taken over by pUL26C but with reduced efficiency as well as loss of an apparent core structure in the resulting capsids [22]. Proteases of several herpesviruses, such as human cytomegalovirus (HCMV) and Kaposi's sarcoma-associated herpesvirus (KSHV), have one or more internal cleavage sites to regulate activity or promote destabilization of the protease [23–25]. For proteases of PrV, HSV-1 and herpes simplex virus type 2 (HSV-2) no internal cleavage sites have been reported.

Herpesvirus maturational proteases exist in a monomer-dimer equilibrium. Dimers are weakly associated with dissociation constants (K_D_) in the micromolar range [26] and are active, while monomers are almost inactive [27]. It was shown that dimerization of the HCMV assemblin is dependent on protein concentration. The fraction of dimeric protease at 0.2 μM was demonstrated to be ~0.3 increasing to ~0.7 at 4.5 μM [27]. Additionally, dimerization is favored by high concentrations of kosmotropic compounds like glycerol [26,27].

Activity of the herpesvirus protease has to be strictly regulated. The enzyme is expressed as full-length pUL26 in the cytosol of infected cells where its concentration is low and thus the inactive, monomeric form is predominant [28]. The major capsid protein is bound by pUL26.5 and translocated to the nucleus *via* the nuclear localization sequence within pUL26.5 [29]. Since pUL26 encompasses pUL26.5, it also contains this nuclear localization sequence. Therefore, autoproteolytic activity of the protease in the cytosol would prevent its localization to the nucleus. When capsid assembly is completed, the protease has to become active to release the scaffold protein from the capsid and to allow DNA packaging. In the capsids, the concentration of protease is much higher than in the cytosol, thus promoting dimerization [28]. Additionally, it was proposed that the capsid environment itself might enhance proteolytic activity of the protease [30].

Several structures of homologous assemblins of other herpesviruses have been published, revealing the overall fold, active site and biological assembly [31–38]. The sequence identities of these structures to the PrV assemblin range from 60% (alphaherpesviruses) to 30% (beta- and gammaherpesviruses) [39,40]. All assemblins consist of 6–9 α-helices surrounding a β-barrel formed by two β-sheets. The catalytic triad is unique among the serine proteases and consists of one serine and two histidine residues. The active site is solvent accessible and distal to the dimer interface. Nevertheless, dimerization drastically influences the activity [27]. There is evidence, that upon dimerization the oxyanion hole is formed by structural changes of a loop containing two strictly conserved arginine residues [41,42].

Currently, crystal structures are available for native dimeric and covalently inhibited dimeric assemblins. Recently, three structures of the truncated KSHV assemblin in complex with helical-peptide mimetics were published [43,44]. These compounds bind to the dimerization area and disrupt the dimerization interface of full-length KSHV assemblin [45]. Here, we report crystal structure analyses of the active dimer, the diisopropyl phosphate-inhibited dimer as well as the non-inhibited monomer of pUL26N from PrV (224 amino-acid residues). The latter is the first-ever structure of an assemblin in its native monomeric state. Comparison of the monomeric and dimeric forms provides insight into the regulation of protease activity by dimerization and a structural basis for rational design of therapeutic substances that trap the protein in the inactive monomeric state.

Additional details and information about herpesvirus proteases and its involvement in capsid maturation are reviewed elsewhere [21,46–52].

## Results and Discussion

### Native and inhibited dimeric pUL26N

#### Overall fold and dimer interface

**Fig 2:**
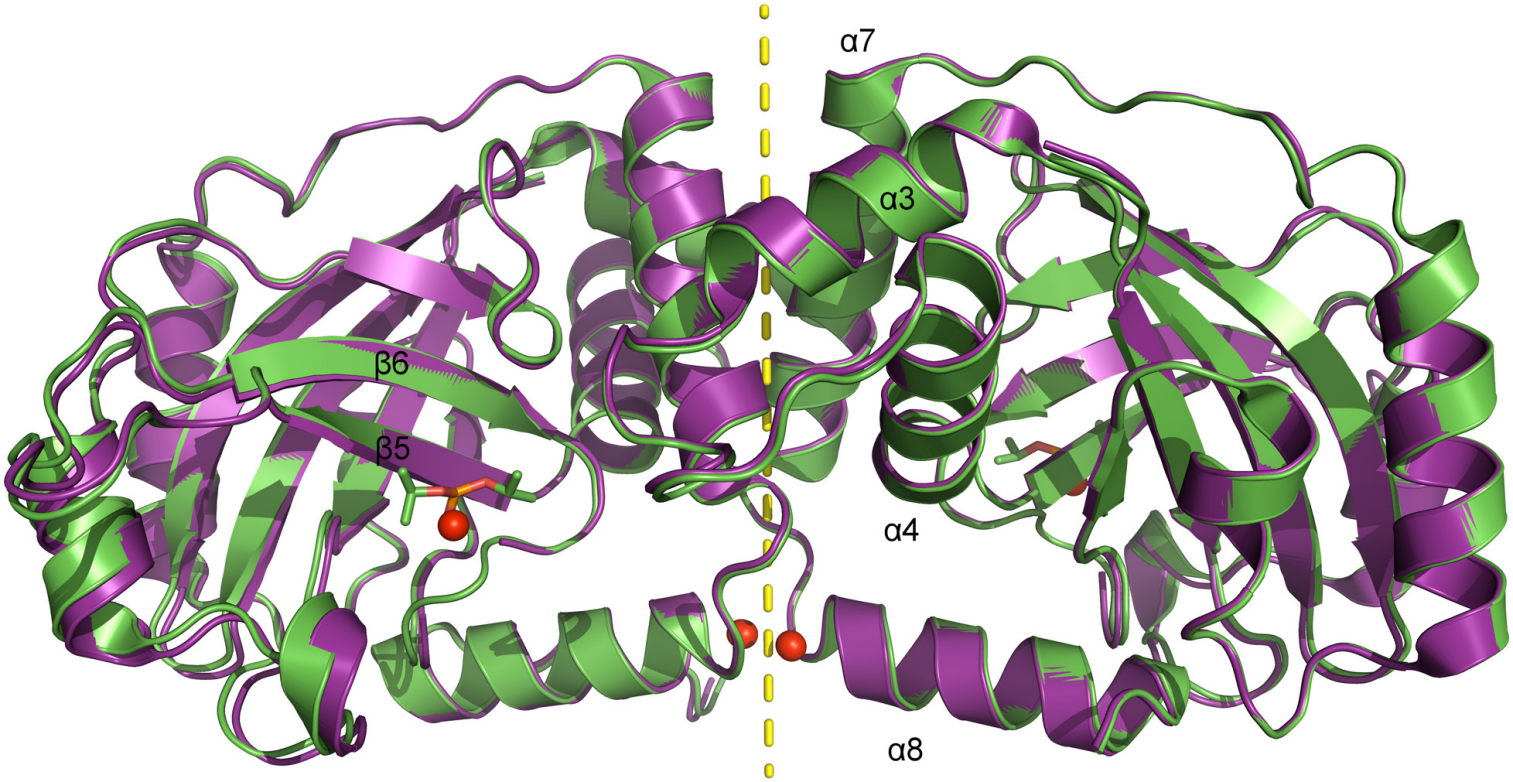
Superposition of dimeric pUL26N from PrV with and without bound inhibitor. Superposition of native (pdb entry 4v07, shown in purple) and diisopropyl fluorophosphate-inhibited (pdb entry 4v08, shown in green) dimeric pUL26N from PrV. Diisopropyl phosphate is shown as stick model and chloride ions as spheres. The two-fold axis of the dimer is shown as a yellow dashed line.

The tertiary structure of pUL26N is a β-barrel consisting of a four-stranded antiparallel and a three-stranded mixed β-sheet (Fig 2) surrounded by eight α-helices. The quaternary structure is a homodimer related by a two-fold non-crystallographic axis with an interface area of 1,273 Å^2^ (native dimer) and 1,258 Å^2^ (inhibited dimer) comparable to all previously published structures (~1,300 Å^2^, determined by PDBePISA [53]). The native and inhibited dimeric structures of pUL26N are nearly identical with an r.m.s. deviation of 0.39 Å (437 aligned Cα atoms, secondary-structure matching [54]) over the whole dimer (Fig 2). The covalently bound diisopropyl phosphate replaces a chloride ion at the active site serine of the native structure. B-factors of the Cα atoms are low throughout the β-barrel and dimer interface and are increased at the far side of the dimer interface (S1 Fig). Overall, B-factors in the inhibited dimeric structure are lower than in the native form (Table 1). This is most likely due to stabilization by inhibitor binding. The overall fold of dimeric pUL26N is similar to homologous structures [31–37]. A superposition of structures of representative assemblins from all herpesvirus subfamilies is shown in S2 Fig. The r.m.s. deviations range from 1.32 Å (pdb entry 1vzv, 410 aligned Cα atoms) to 2.58 Å (pdb entry 1cmv, 334 aligned Cα atoms) over the whole dimers.

**Table 1:** Summary of data collection and refinement statistics.

Data collection statistics¹	Monomer	Native dimer	Inhibited dimer
X-ray source	BESSY II/BL14.1	BESSY II/BL14.1	BESSY II/BL14.1
Wavelength (Å)	0.91841	0.91841	0.91841
Temperature (K)	100	100	100
Space group	P2_1_2_1_2_1_	P2_1_2_1_2_1_	P2_1_2_1_2_1_
a/b/c (Å)	40.17/98.61/131.11	51.38/75.98/110.76	52.06/76.23/111.05
Max. resolution (Å)	2.05 (2.16–2.05)	2.10 (2.21–2.10)	2.03 (2.14–2.03)
Unique reflections	33,525	25,947	29,319
Redundancy	6.5 (6.3)	6.5 (6.7)	6.5 (6.3)
R_meas_ (%)	7.3 (77.4)	7.8 (88.5)	11.5 (82.1)
Mean I/σ(I)	16.3 (2.7)	19.3 (2.4)	13.1 (2.2)
Completeness (%)	99.6 (97.8)	99.8 (99.2)	99.9 (99.4)
Wilson B-factor (Å^2^)	44.0	44.7	35.0
Solvent content (%)	49.6	39.5	40.6
**Refinement statistics**			
Resolution (Å)	78.8–2.05	62.7–2.10	62.8–2.03
No. of reflections	33,525	25,947	29,319
R/R_free²_ (%)	17.9/22.7	17.8/23.7	17.0/23.4
Figure of merit	0.837	0.791	0.825
Protein residues/water molecules	435/148	442/133	437/263
R.m.s.d. of bond lengths (Å)	0.019	0.015	0.016
R.m.s.d. of bond angles (°)	1.993	1.722	1.762
Average B-factor (Å^2^)	52.0	49.0	36.0
**Ramachandran statistics** **³** **(%)**			
Most favored regions	96.55	97.06	96.80
Outliers	0	0	0.23

^¹^ Numbers in parentheses refer to the highest resolution shell.

^²^ R = Σ||F_o_-|F_c_||/Σ|F_o_|, where F_o_ and F_c_ are the observed and calculated structure factors, respectively. R_free_ = analogous to R-factor except the summation is over 5% of reflections excluded for refinement.

^³^ Categories were defined using MolProbity [55].

The dimer interface is formed by helices α3, α4 and α7 establishing a hydrophobic patch towards the adjacent monomer like the corresponding α-helices in the structures of assemblins from varicella zoster virus (VZV, pdb entry 1vzv) and HSV-2 (pdb entry 1at3). As already described before, dimer interfaces of alphaherpesvirus assemblins are slightly different from those of beta- and gammaherpesvirus assemblins. In dimers of beta- and gammaherpesvirus assemblins, the helices corresponding to helix α7 of dimeric PrV assemblin are nearly parallel, whereas in alphaherpesvirus assemblins they form an angle of 30–40° [35]. A difference to all other published structures is the loop α3-α4 in PrV pUL26N that forms one α-helical turn in the other structures, except for HSV-2 assemblin where this part is not modeled. Moreover, the N-termini of helices α8 of PrV pUL26N point towards each other with the positive dipole moment compensated by one chloride ion per monomer. The presence of such chloride ions has not been observed in previous structures. Probably, the solvent interaction is sufficient to compensate for the positive dipole moment in dimers of beta- and gammaherpesvirus assemblins.

### Active site and oxyanion hole

**Fig 3:**
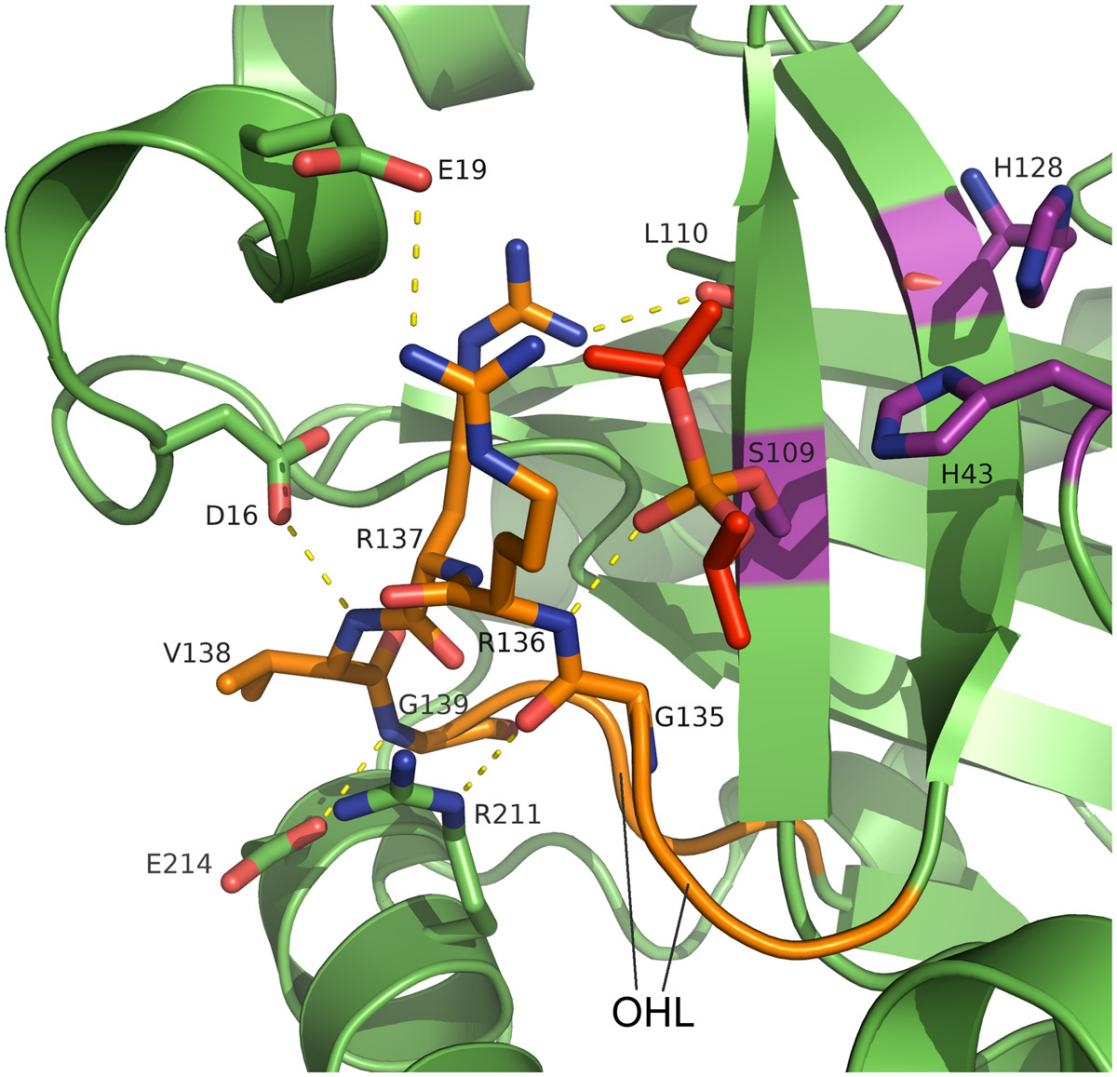
Active site and oxyanion hole loop of inhibited dimeric pUL26N from PrV. A detailed view of the active site and the hydrogen-bond network of the oxyanion hole is shown. The oxyanion-hole loop is colored orange, the covalently bound diisopropyl phosphate is colored red and the catalytic triad is colored purple. Dashed lines illustrate parts of the hydrogen bond network (distances in the range of 2.7–3.4 Å). The strictly conserved Arg136 forms the oxyanion hole with its peptide backbone N-H. Diisopropyl phosphate mimics the transition state of the natural substrate.

The active site serine is solvent accessible and the catalytic residues are part of β-strands β5 (Ser109) and β6 (His128) and the β2-β3 loop (His43), like in earlier structures [31–37]. In our inhibitor complex, covalent binding of diisopropyl fluorophosphate has formed a phosphate ester with the active-site serine in a manner also observed in crystal structures of homologous assemblins [34]. In the native structure, a chloride ion occupies the oxyanion hole, which is formed by the β6-β7 loop (residues 133–142, further referred to as oxyanion-hole loop, OHL, Fig 3). Two consecutive arginine residues in this loop (Arg136 and Arg137) are strictly conserved throughout all herpesvirus maturational proteases (S3 Fig). The backbone N-H of Arg136 provides the first hydrogen-bond donor of the oxyanion hole. A water molecule as second hydrogen-bond donor supports anion stabilization, as does the positive local environment established by these two arginine residues. For HCMV assemblin it was shown that this water molecule plays a role in catalysis [56]. It is kept in place by a second water molecule that is positioned by hydrogen bonds of the peptide backbone oxygen of Leu10 (loop β1-α1) and the peptide N-H of Leu110 (β5). Both water molecules are present in the dimeric structures of assemblins from PrV (this report), HSV-2 (pdb entry 1at3), KSHV (pdb entries 1fl1, and 2pbk) and HCMV (pdb entries 1cmv, 1wpo, 1id4, 1iec, 1ied, 1ief, and 1ieg). The positions of these water molecules seem to be conserved in all active assemblins. Our findings for the composition of the oxyanion hole are consistent with those reported for HSV-2 [34].

An extensive network of hydrogen bonds stabilizes the OHL (S4 Fig). This network includes conserved water molecules and parts of the α1 region, α8 and β5. There are five strictly conserved residues in the OHL and each of these is involved in the network of hydrogen bonds that positions Arg136 and stabilizes the oxyanion hole.

The side chain of Arg137 forms hydrogen bonds to the peptide backbone of Leu20 (α1) and Leu110 (β5), which are also conserved in assemblins (S3 Fig). The OH-group of the conserved Thr140 forms a hydrogen bond to the backbone oxygen of Arg137 whereas the backbone oxygen of Thr140 accepts a hydrogen bond from the backbone N-H of Ala108 (β5). Inspection of other assemblin structures showed that these hydrogen bonds are present in all dimeric structures. In the PrV assemblin, the peptide oxygen of Val138 is connected to the peptide N-H of Leu12 *via* hydrogen bonds mediated by a conserved water molecule (found in HSV-2 and KSHV assemblins). Most likely this water molecule and hydrogen bonding pattern are present in all herpesvirus maturational proteases.

Additionally, the OHL of PrV pUL26N is maintained by hydrogen bonds of the side chains of Asp16 (α1), Glu214 and Arg211 (both from α8) with the peptide backbone of Val138, Gly139 and Gly135, respectively. These hydrogen bonds are also present in HSV-2 assemblin with Glu214 replaced by Gln. Identical residues are present in the VZV assemblin, but the side chains of Glu and Asp point in different directions and do not form the corresponding hydrogen bonds. This ambiguity may result from the limited resolution of that structural model (pdb entry 1vzv with a resolution of 3 Å, no structure factors were deposited). Most likely, the loop is stabilized comparable to HSV-2 and PrV assemblins.

### Monomeric pUL26N

Diffraction datasets of dimeric pUL26N were collected from needle-shaped crystals and phasing by molecular replacement was successful either by using a monomer or the complete dimer as search models. For datasets derived from morphologically different, plate-shaped crystals, molecular replacement was only successful when using a monomer as a search model. In the resulting structure, two chains are present in the asymmetric unit. These chains and their adjacent symmetry mates are not in a proper position to form the known dimer. PDBePISA [53] suggests one assembly. This putative dimer has no local dyad, which is unusual for biologically relevant dimers [57,58]. The interface area (978 Å^2^) is much smaller than average for homodimers of this molecular weight (~1,500 Å^2^) [57]. Furthermore, the helices forming the interface have B-factors (60–100 Å^2^) above average (52 Å^2^). Thus, the suggested dimeric interaction is a mere crystal contact and the crystal structure actually corresponds to monomeric pUL26N.

**Fig 4:**
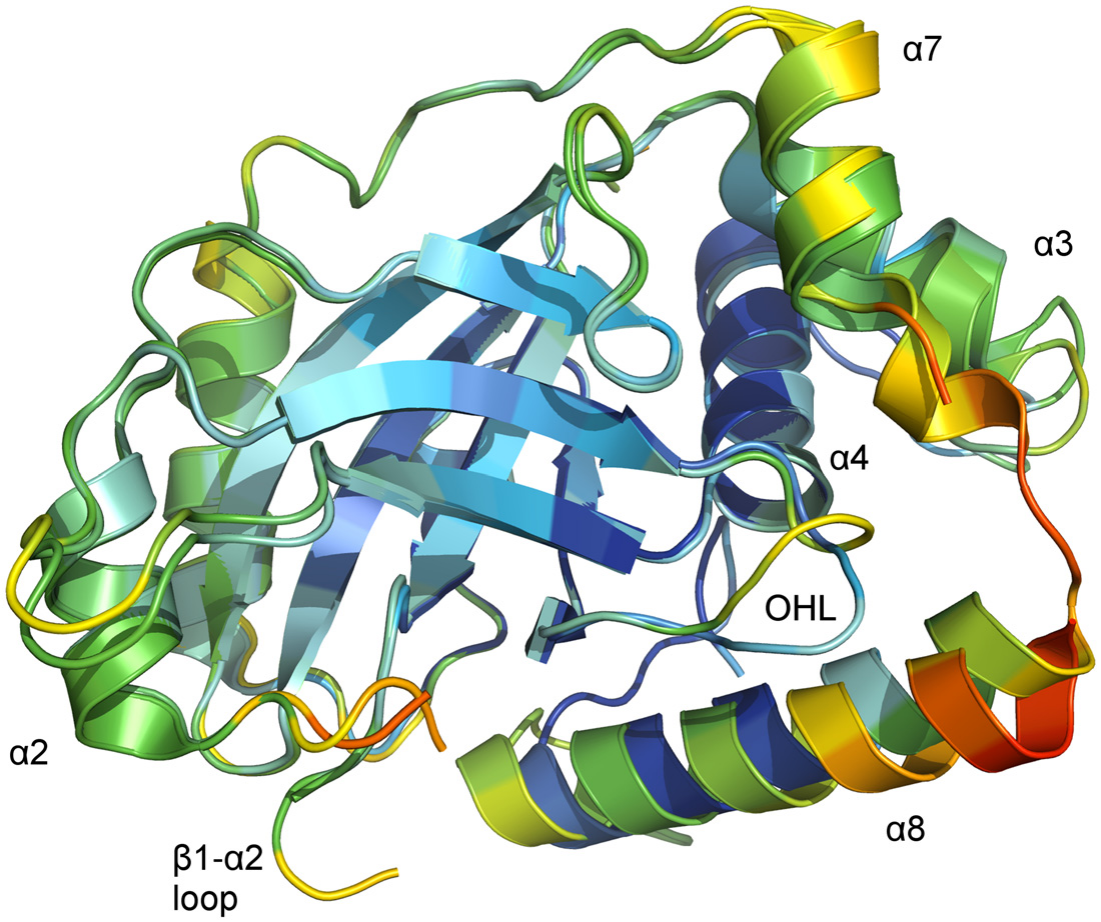
B-factor coloring of superposed chains of the asymmetric unit of monomeric pUL26N from PrV. Superposition of chain A and chain B of monomeric PrV pUL26N (pdb entry 4v0t) with Cα atom B-factor coloring. B-factors below 40 Å^2^ are colored deep blue. B-factors above 100 Å² are colored orange or red. The OHL and the dimerization helices that differ in both chains are labeled. The β-barrel and the far side of the dimerization area show low to moderate B-factors. High B-factors are observed for the α7-α8 loop and the partial disordered loop β1-α2.

The region corresponding to helix α1 in dimeric PrV pUL26N is disordered in the monomeric form. For ease of comparison, in this report numbering of the helices in the monomeric protease is adapted to that of the dimeric protease. The β-barrel and distal side of the dimerization area of chain A align very well with chain B of the asymmetric unit. The OHL and helices α3, α7 and α8 of the dimerization area, on the other hand, differ in chain A and B (Fig 4). The OHL has two alternative conformations in chain A with equal occupancies. One conformation correlates to the OHL of chain B and the other one is shifted towards α8 due to crystal contacts. Compared to chain B the helices α3 and α7 of chain A are bent and helix α8 is slightly tilted. Accordingly, the overall r.m.s. deviation of chain A and B is 1.03 Å (207 aligned Cα atoms). Furthermore, there is no electron density observed for residues 16–19 of chain A (β1-α2 loop) and very weak electron density for residues 14–18 and 194–196 of chain B (β1-α2 loop and the short α7-α8 loop, respectively).

The core of the monomer is rigid as indicated by low B-factors of Cα atoms in the β-barrel, helix α4 and most of helix α8 of chain A (Fig 4). In contrast, the periphery of both monomers is flexible as evidenced by increased B-factors of Cα atoms at the distal side of the dimerization area, the OHL, and the dimerization helices α3 and α7 as well as in helix α8 of chain B. B-factors around 100 Å^2^ are also observed at the N-termini of helices α8 in both chains.

Taken together, these observations show that the dimerization area and the parts necessary for formation of the oxyanion hole in dimeric pUL26N are flexible and not strictly ordered in monomeric pUL26N.

### Comparison of monomeric and dimeric pUL26N from PrV

**Fig 5:**
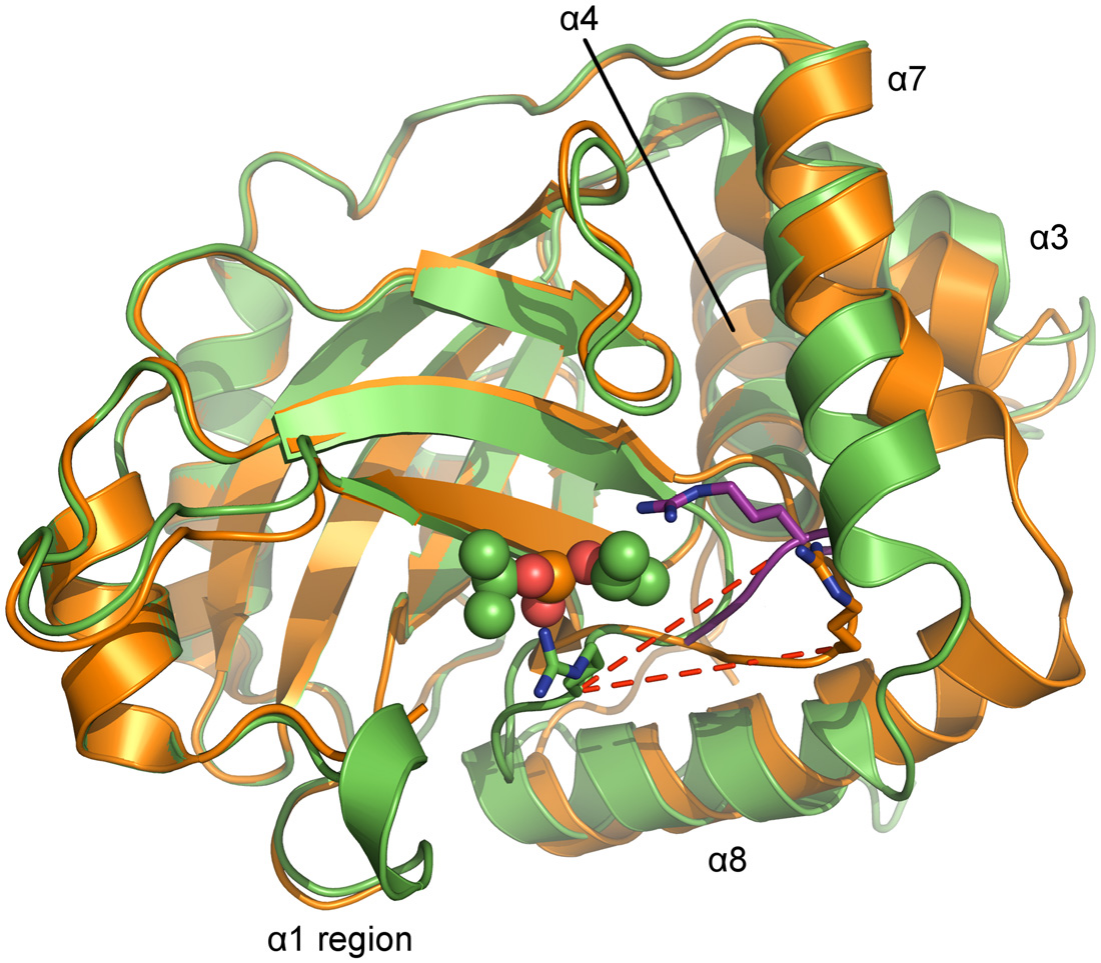
Superposition of monomers of monomeric and dimeric PrV assemblin. Superposition of chain A of monomeric (shown in orange, alternative conformation of the oxyanion-hole loop colored purple) and chain B of inhibited dimeric (shown in green) pUL26N from PrV. Arg136 (stick model) of the oxyanion-hole loop moves ~11 Å (red dashed lines) to form the oxyanion hole. The helices, which undergo significant changes upon dimerization, are labeled. Diisopropyl phosphate is represented as space-filling model.

The tertiary structures of monomeric and dimeric PrV pUL26N are partially similar. The r.m.s. deviations of inhibited dimeric pUL26N chain B with chain A and chain B of monomeric protease are 0.93 Å (192 aligned Cα atoms) and 1.01 Å (190 aligned Cα atoms), respectively. The β-barrel, the distal side of the dimer interface, and helix α4 of the dimer interface are almost identical in monomeric and dimeric pUL26N (Fig 5). The r.m.s. deviations of these parts of inhibited dimeric pUL26N chain B with chain A and chain B of monomeric protease are 0.54 Å (151 aligned Cα atoms) and 0.63 Å (149 aligned Cα atoms), respectively. In contrast, significant differences are observed for the OHL, N- and C-termini of helices α3, α7 and α8 of the dimer interface, and the α7-α8 loop (Fig 5).

In the monomer, the N-terminus of helix α8 is one and a half turn longer, but the C-terminus of helix α7 is one turn shorter. Thus, the α7-α8 loop conformation is shifted towards α8 upon dimer formation. In dimeric pUL26N helices α3 and α7 form a hydrophobic cleft that is occupied by helix α7 of the second monomer within the dimer. This cleft is closed in the monomer by bending the C-termini of these helices towards each other (Fig 5).

**Fig 6:**
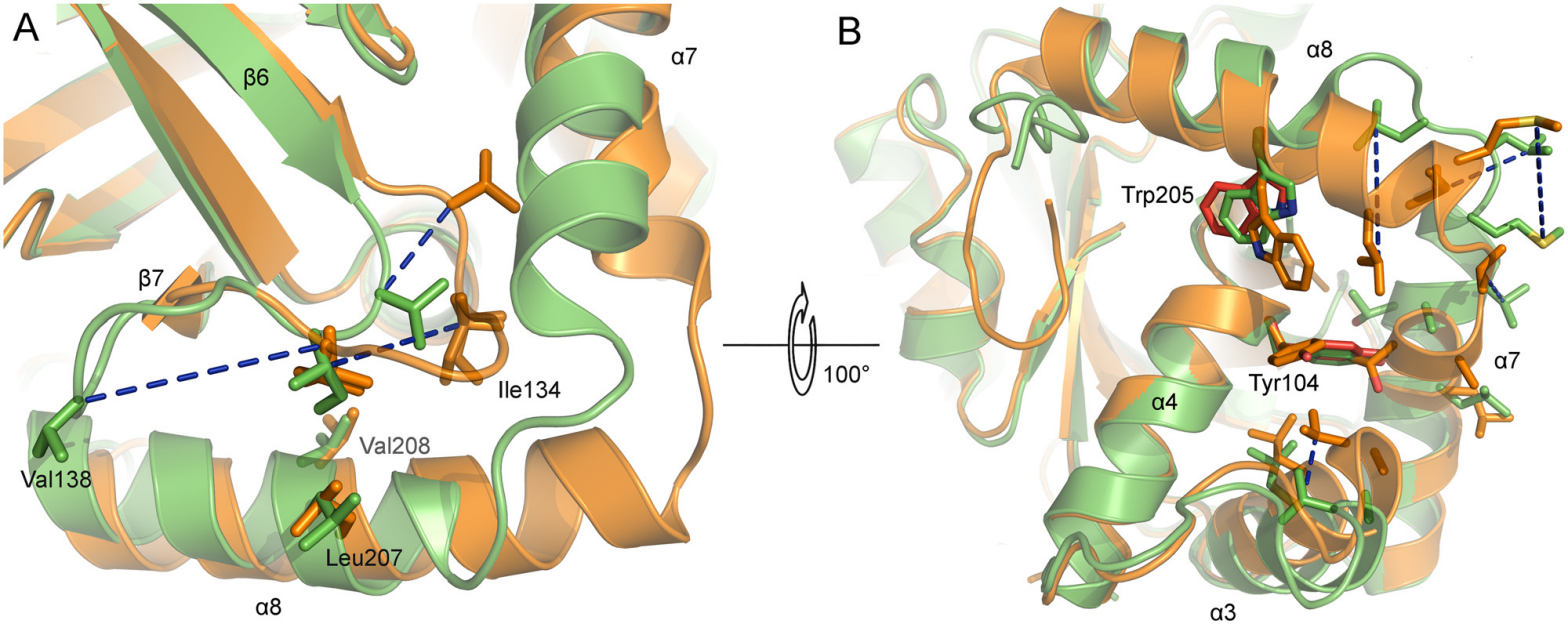
Active site and dimer interface helices of superposed monomers of monomeric and dimeric PrV assemblin. Superposed A-chains of monomeric (shown in orange) and inhibited dimeric (shown in green) PrV protease. Blue dashed lines connect corresponding residues of both structures. (A) Hydrophobic residues of the oxyanion-hole loop (OHL, only one conformation shown). (B) Hydrophobic residues of α3, α7 and α8, which undergo changes, are shown. The OHL is omitted for clarity. Close to the OHL and the dimerization area the residues Tyr104 (α4) and Trp205 (α8) (both colored red) have different side-chain rotamers in chain A and B, due to crystal contacts of chain A indicating the not established dimer interface. In monomeric protease, the helices α3 and α7 move towards each other. After dimer formation, this space is occupied by α7 of the second monomer (not shown). Hydrophobic residues of helices of the dimerization area in monomeric protease are clustering together to be less exposed to solvent. In the dimeric form, these residues are enabling dimerization.

In the monomeric pUL26N, the OHL is positioned near the N-terminus of α8. Thus, the activation of pUL26N relies on dimerization-induced allosteric changes, which shift the OHL towards the active site (Fig 6A). The top of the OHL (Arg136Cα) moves approximately 11 Å. In the dimer, the N-terminus of α8 is unwound, so some residues which point towards the protein core in the monomeric pUL26N are buried by the adjacent monomer of the dimer (Fig 6B). However, the side chain positions of the catalytic triad are unchanged as suspected by Batra *et al*. [41].

Comparison of the active and inactive conformations of the OHL reveals a key location in the structure that is occupied by alternative hydrophobic residues. In dimeric PrV pUL26N, Ile134 is present at this position, whereas in the monomeric form Val138 takes its place (Fig 6A). The hydrophobic character of the Ile134 position is highly conserved in all sequences and three-dimensional structures of assemblins (S3 Fig). The hydrophobic character of Val138 is type-conserved in alphaherpesvirus sequences. Both residues are kept in place by hydrophobic interactions with two side chains of helix α8 (Leu207, Val208) or the corresponding helices in related structures. The hydrophobic character of these residues is conserved in herpesvirus proteases, although Thr and long, partially aliphatic side chains appear to be tolerated (e.g. Thr in Epstein-Barr virus (EBV) assemblin, or Lys in KSHV assemblin, S3 Fig).

Another consequence of the different conformation of the OHL in the monomeric form is that the Asp16-containing region and α1 are disordered. This loop, the OHL and major parts of the dimer interface area are flexible in monomeric pUL26N as indicated by partially disordered segments and high B-factors. Thus, the crystallographically observed increase of order upon dimerization of PrV assemblin is in line with the disorder-to-order mechanism of dimerization previously proposed for KSHV assemblin [28,43].

### Impact of a C-terminal deletion

In our crystallization assays we used a construct of PrV pUL26N that was shortened by one C-terminal amino-acid residue (Ala225) compared to the *in vivo* form. The resulting overall fold, conformation of the OHL, and position and orientation of the residues of the catalytic triad are identical to those in previously determined dimeric full-length assemblin structures of related herpesviruses, strongly suggesting that the C-terminal deletion does not affect the activity or structure of the protease. Moreover, native and inhibited dimers of PrV pUL26N crystallized isomorphously and the diisopropyl phosphate-ester of the active-site serine is quantitatively observed in the inhibited structure.

The inhibitor diisopropyl fluorophosphate reacts specifically with the active-site serine, but hydrolyzes in aqueous solutions with a half-life of one hour at pH 7.5 and even faster at higher pH [59]. After incubation of PrV pUL26N with a sevenfold excess of diisopropyl fluorophosphate (5 mM) in a buffer at pH 7.5 at room temperature for one hour, the protein was crystallized at pH 8. Based on these constraints quantitative inhibition of PrV pUL26N (minimum 90% in the crystal) prior to inhibitor hydrolysis can be calculated to occur at a second order reaction rate of at least 0.09 s^−1^ M^−1^. This is consistent with the known reaction rate of full-length HSV-1 assemblin with diisopropyl fluorophosphate of 1 s^−1^ M^−1^ at higher pH (pH 8.0) and higher temperature (30°C) [60]. Thus, the catalytic site of PrV pUL26N remains fully reactive with respect to this substrate-like inhibitor. Furthermore, earlier work has shown that proteolytic activity with respect to substrates containing the M-site is not significantly reduced by 3- or 8-residue C-terminal truncations [61]. C-terminally extended assemblins on the other hand do show a considerable decrease in activity [37,61,62], presumably because such an extension sterically interferes with the proper positioning of helix α8, which is required to establish the correct conformation of the OHL. The absence of Ala225, however, does not interfere in any way with helix α8, the conformation of the active site, and the OHL. Indeed, in the crystal structure of VZV assemblin the C-termini are disordered [35], further confirming that the C-terminal region does not have a substantial impact on the overall protein fold.

### Monomer-dimer equilibrium

Structural properties and self-association behavior of PrV pUL26N in solution were characterized by small-angle X-ray scattering (SAXS). Data were recorded at protein concentrations between 0.5 mg/ml and 10 mg/ml. Normalized SAXS intensities vary with protein concentration, suggesting the presence of more than one oligomerization state. The experimental SAXS curves can be accurately described assuming a monomer-dimer equilibrium and using form factors for the individual states derived from the monomeric and dimeric crystallographic models (a representative example is shown in S5 Fig). Monomer volume fractions resulting from curve fitting with the program OLIGOMER [63] are shown in S5 Fig and S1 Table. At the highest protein concentration investigated (10 mg/ml, which approximately corresponds to the starting concentration in our crystallization experiments), a considerable volume fraction (30%) of pUL26N is present in the monomeric form. This observation is consistent with the fact that crystals of the monomer could be obtained under these conditions. In contrast, inclusion of 0.2 M MgCl_2_ in the buffer used for SAXS measurements markedly shifts the monomer-dimer equilibrium, resulting in nearly complete dimerization at a protein concentration of 10 mg/ml. This result may explain why crystallization of the dimeric form of pUL26N required the presence of MgCl_2_. Particle shape under conditions that result in virtually complete dimerization according to the analysis with OLIGOMER (*i*.*e*. 10 mg/ml protein in the presence of MgCl_2_) was also determined independently by means of an *ab initio* approach. The size and oblong shape of the final bead model obtained with DAMMIN [64] are in good agreement with the crystallographic structure of the pUL26N dimer (S5 Fig), further confirming the nature of the concentration-dependent self-association that is observed here. The dissociation constants with and without MgCl_2_ can be estimated to 200 μM and 50 μM, respectively (S5 Fig). Higher oligomers than dimers are not observed.

### Monomeric PrV pUL26N compared to truncated KSHV assemblin in complex with helical-peptide mimetics

Three structures of KSHV assemblin (KA) with helical-peptide mimetics (HPMs) have been published to date with pdb entries 3njq, 4p2t, and 4p3h [43,44]. These HPMs disrupt dimer formation in full-length KA as shown by size-exclusion chromatography [45]. In solution, both C-terminal helices of the monomeric KA are unfolded according to NMR- and CD-spectroscopic data [28,43]. Therefore, 34 C-terminal residues of KA were truncated for crystallization of these HPM complexes. One of these truncated helices is the major dimer-interface helix, so this truncated KA is obligate monomeric. Consequently, the models of these HPM complexes were stated as monomeric [43,44]. Inspection of these models led us to the conclusion, that the HPMs function as an artificial dimer interface for this truncated KA. PDBePISA suggests dimers or higher association states for HPM complexes (buried surface area of ~1,200 Å^2^ per monomer). The monomer is termed as unstable in solution, because the hydrophobic HPMs would be heavily solvent exposed. Compared to the native dimeric structure of full-length KA, one monomer is rotated approximately 80° around an axis roughly perpendicular to the dimer interface in the artificial, inactive HPM complexes of truncated KA (S6 Fig).

Although artificial, the HPM-interface underlines the importance of hydrophobic interfaces as suitable drug targets. The used HPMs were reported to bind to assemblin dimer interfaces of all herpesvirus classes. The IC_50_-values for these HPMs against the representative alphaherpesvirus assemblin (HSV-2 assemblin) were significantly higher than those against HCMV, EBV and KSHV assemblins, indicating much weaker binding to HSV-2 assemblin [44]. The C-terminal helices of alphaherpesvirus assemblins are likely ordered due to the hydrophobic key position being occupied by alternative conserved hydrophobic residues of the OHL. Thus, the HPMs have to compete against the C-terminal helices for binding which explains the observed higher IC_50_-values. Additionally, the most important residues for dimerization in KA, the so-called “hot spot” residues [65], are Trp109 (α4) [43] and maybe Phe76 (α3). These correspond to Tyr (α4) and Leu (α3), respectively, in PrV, VZV, HSV-1 and HSV-2 assemblins (S3 Fig). Neither Leu nor Tyr are considered as “hot spot” residues [65] and thus, binding of these HPMs is likely weakened. Screening for suitable mimetics is necessary to achieve specific and efficient inactivation of alphaherpesvirus proteases.

As mentioned above the HPMs force an artificial assembly of truncated KA in contrast to our native monomeric PrV assemblin. Therefore, a detailed comparison of monomeric PrV assemblin and HPM complexes of truncated KA is discussed in the supplement (S1 Text and S7–S10 Figs).

### Inspection of a putative cation binding-site

A putative cation was found in both dimeric structures from PrV. A distorted octahedral arrangement of the coordinated water molecules with mean distances of 2.1 Å strongly suggests the presence of a cation rather than water. Since the crystallization buffers contain MgCl_2_, it is highly probable that these cations are Mg^2+^ ions. For verification, divalent cations with higher electron density were tested in the crystallization procedure. Mn^2+^ was able to substitute for Mg^2+^ but the resulting crystals diffracted considerably less well. The best dataset that we managed to collect at a wavelength near the Mn absorption edge had a resolution of 3 Å, but no significant anomalous signal was detected and electron density at the putative Mg^2+^/Mn^2+^ position was too weak to unambiguously confirm the presence of a Mn^2+^ ion. Presumably, the presence of putative Mg^2+^ ions in the structures is a direct result of the crystallization conditions (containing 400 mM MgCl_2_) and does not reflect functional Mg^2+^ binding by the native protein since the metal ions compensate negative charges from two adjacent dimers, rather than within one dimer (S11 Fig). Thus, Mg^2+^ ions or divalent cations with related properties are presumably necessary for the observed crystal packing of dimeric pUL26N from PrV.

### Presumable temperature-sensitive pUL26 phenotypes

**Fig 7:**
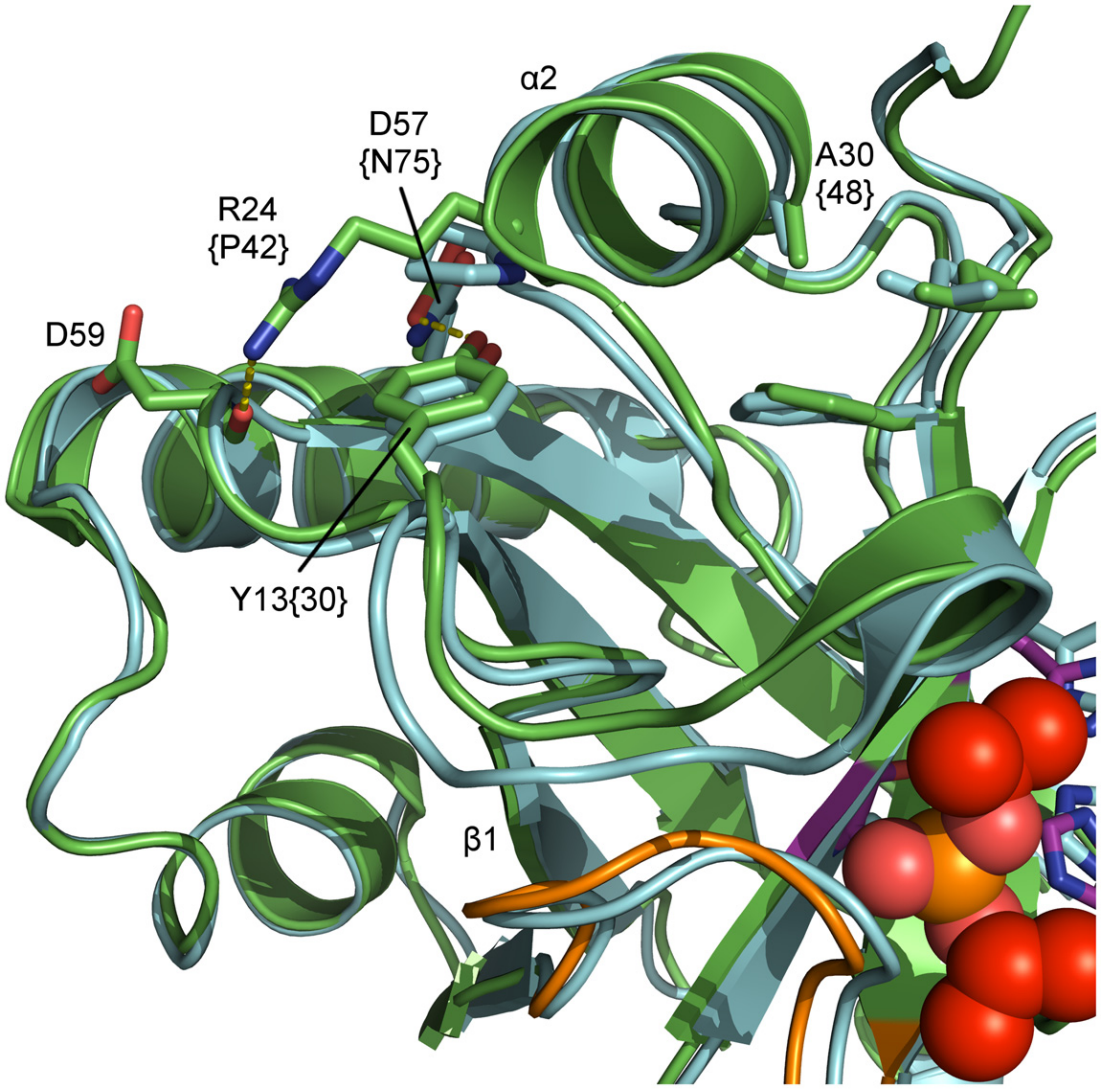
Superposition of inhibited dimeric pUL26N from PrV and HSV-2. Detailed view of relevant residues for the proposed temperature-sensitive (ts) variant of pUL26N from PrV. Shown are a superposition of inhibited dimeric PrV pUL26N (colored green) and HSV-2 (pdb entry 1at3, colored cyan). Residues for the temperature-sensitive phenotype of HSV-2 assemblin (identifier in braces), the proposed residues for ts mutant of PrV assemblin and interaction partners are labeled. The oxyanion-hole loop (OHL) is shown in orange, diisopropyl phosphate in red and the catalytic triad in purple. The polypeptide around residues 15–21, including helix α1, is likely disordered at the non-permissive temperature. This results in destabilization of the OHL and reduced activity.

For HSV-1 protease a temperature-sensitive (ts) phenotype was described by mutating Y30F and A48V [66]. Mutating the corresponding residues in PrV protease (Y13F/A30V), however, did not result in the desired phenotype. Hence, these mutations may not sufficiently destabilize PrV pUL26N. In HSV-1 protease, it is highly probable that the result of these mutations is a destabilization of the region around helix α1. This helix and surrounding loops stabilize the OHL in the active conformation. In the PrV assemblin Arg24 is located at the N-terminus of α2. This residue stabilizes the α1 region, because its side chain forms a hydrogen bond to the peptide oxygen of Asp59 (Fig 7). This hydrogen bond is missing in HSV-1 assemblin, since the corresponding residue is Pro42. Consequently, we propose a Y13F/R24P/A30V mutant for achieving the desired ts phenotype in PrV assemblin. If the additional R24P exchange completely inactivates the protease, an R24K variant could also be taken into account. The slightly shorter side chain of Lys may cause a varied hydrogen-bond network and a weaker hydrogen bond to the peptide oxygen of Asp59.

### Conclusions

During capsid assembly, pUL26 accumulates in the nascent capsids because of its C-terminal scaffold-protein part. Thus, the local high concentration of the protease is promoting dimerization and autoproteolysis occurs to release the scaffold from capsids for DNA packaging. In comparison with its dimeric structure, the monomer of pUL26N reveals changes at the dimerization area, in line with allosteric changes of a loop forming the oxyanion hole. As previously anticipated, the core of the protease including the positions and orientation of the active-site residues remains unchanged, but the oxyanion hole is disrupted in the monomeric form [41,42]. The monomeric structure presented here is not truncated and does not contain any inhibitors. Thus, it constitutes the first reliable model for native monomeric structures of other assemblins.

Dimerization induces the following allosteric events: helix α7 of a monomer interleaves between helices α3 and α7 of a second monomer moving these helices farther apart. At the same time, helix α7 is elongated by one turn at its C-terminus to the cost of one N-terminal turn of helix α8. This allosteric process forces the OHL to shift to the vicinity of the active-site serine and builds a far-reaching network of hydrogen bonds with side chains of helix α8 and the polypeptide of strand β5. Furthermore, residues 13–20 of the β1-α2 loop become ordered and form helix α1 in the dimer by getting involved in that network of hydrogen bonds. In this position, the OHL forms the oxyanion hole and activity of the protease is established. The extent of disorder at the dimerization area will vary in assemblins of different herpesviruses, but the general disorder-to-order mechanism of dimerization [28,43] will very likely hold for all assemblins.

The molecular structure of dimeric pUL26N will help to engineer a temperature-sensitive phenotype of the PrV protease. A temperature-sensitive variant will be a powerful tool to observe subsequent steps of viral replication in a synchronous wave [19]. This will provide valuable data on kinetics for cleavage, packaging of the DNA, nuclear egress and intracellular trafficking of the virions.

The structure of monomeric PrV assemblin is the paradigm for monomeric states of other assemblins, primarily from alphaherpesviruses. Detailed knowledge of this structure, conformational changes and sequence specific contacts upon dimerization are a rational basis for the development of drugs binding to the dimerization area and, thus, trapping the inactive monomeric state [45]. Inhibition of dimerization suppresses protease activity and therefore prevents the assembly of fully functional virus capsids.

Small-angle X-ray scattering (SAXS) of PrV assemblin in solution revealed that dimerization increases with protein concentration and in the presence of MgCl_2_. Dissociation constants in the micromolar range are comparable to those observed for other assemblins [26,27]. Divalent cations like Mg^2+^ or Mn^2+^ are required for crystallization of the dimeric PrV assemblin, because these cations support crystal packing by compensating negative charges of neighboring dimers. Since MgCl_2_ shifts the condition of equilibrium towards dimeric pUL26N, the concentration of the monomeric form is likely below the critical nucleation concentration resulting in crystals of the dimeric form only. Accordingly, crystallization of the monomeric assemblin requires absence of divalent cations. The monomeric fraction of ~0.3 is sufficient for nucleation and the monomer-dimer equilibrium provides a steady supply of monomeric pUL26N. The monomeric form is increasingly favored since crystallization of monomeric pUL26N decreases the concentration of pUL26N in solution. Similar cases with a minor monomeric fraction crystallizing from a monomer-dimer equilibrium were reported earlier [67].

## Materials and Methods

### Expression and purification

Full-length pUL26 cleaves itself at two positions, and therefore expression of the full-length protein leads to an inhomogeneous product that is unlikely to crystallize. Accordingly, cleavage was prevented by cloning a stop codon behind Gln224. The resulting coding sequence contains the protease fraction of pUL26 (pUL26N) only.

N-terminally (His)_6_-tagged pUL26N was expressed using a pET28a+ vector in *E*.*coli* strain BL-21 (DE3). Cells were grown in LB medium to an OD_600_ of 0.5–0.8 at 37°C and then induced by addition of isopropyl β-D-1-thiogalactopyranoside to a final concentration of 1 mM. Cells were lysed by sonication. The protein was purified by performing immobilized metal-ion affinity chromatography using a Poros MC 20 column loaded with Ni^2+^ ions (0.5 M NaCl, 50 mM Tris/HCl pH 7.5, 5% glycerol, eluted with a gradient of 0–250 mM imidazole). The protein was checked for its purity by SDS-PAGE and then concentrated to ~20 mg/ml. Aliquots were stored at −80°C. The purified protein was not tested for enzymatic activity.

### Crystallization and cryo-protection

Crystals were obtained using the hanging-drop vapor diffusion method at 22°C. First crystals grew in drops containing 1 μl pUL26N concentrate and 1 μl precipitant solution (0.1 M Hepes pH 7.5, 20% PEG 8,000) within several days. The quality and size of the crystals could be increased by optimization of the composition of the precipitant solution and the ratio of protein solution to precipitant solution. The morphology of the crystals changed from plate-shaped to needle-shaped when MgCl_2_ was used as an additive in the crystallization procedure. Plate-shaped crystals turned out to be monomeric pUL26N, whereas the dimer formed needle-shaped crystals. For crystallization with inhibitor, the concentrated protein solution was incubated with a final concentration of 5 mM diisopropyl fluorophosphate for 1 hour prior to crystallization.

Best crystals of native dimeric, inhibited dimeric and monomeric pUL26N grew in drops containing 2 μl pUL26N concentrate and 1 μl precipitant solution. Precipitant solution for monomeric pUL26N consisted of 0.1 M Tris/HCl pH 8, 8% PEG 8,000 and crystals grew within one week. Precipitant solutions for native dimeric and inhibited pUL26N consisted of 0.1 M Tris/HCl pH 8, 14% PEG 8,000, 0.4 M MgCl_2_ and 0.1 M Tris/HCl pH 8, 20% PEG 8,000, 0.2 M MgCl_2_, respectively.

All crystals were cryo-protected by soaking for 15 s in drops of precipitant solution with increasing amounts of PEG 400. Final concentrations of PEG 400 were 25% (monomer), 18% (native dimer) and 17% (inhibited dimer). Cryo-protected crystals were flash-frozen in liquid nitrogen.

### X-ray diffraction, phasing and model refinement

Datasets were measured at 100 K with a PILATUS-6M detector at beamline BL14.1, operated by the Helmholtz-Zentrum Berlin (HZB) at the BESSY II electron storage ring (Berlin-Adlershof, Germany) [68]. 1,800 images were collected at X-ray wavelength 0.91841 Å with an exposure time of 0.5 s and an oscillation range of 0.1°.

All datasets were processed using XDS and Aimless [69–72]. Further details on data collection and processing are listed in Table 1. For determination of the Matthews coefficient and solvent content of the unit cell the CCP4 Program suite (version 6.4.0) was used [73–76].

The structures of monomeric and native dimeric pUL26N were solved *via* molecular replacement using Phaser [77]. The starting model for native dimeric pUL26N was the A-chain of pdb entry 1at3 (the homologous protein from HSV-2) edited with Chainsaw [78,79]. The A-chain of native dimeric pUL26N (pdb entry 4v07) was used as the starting model for monomeric pUL26N. Since both dimeric forms were isomorphous, the inhibited structure could be solved by refining the native structure against the dataset of the inhibited form. Cycles of model building and refinement were carried out using Coot (version 0.7.1) and Refmac5 (version 5.8.0073), respectively [80–88]. Further details on refinement are listed in Table 1. The oligomerization states were confirmed by PDBePISA [53]. All figures representing structural models were prepared using PyMOL version 1.7.1.3 [89].

The diffraction data and refined models of monomeric, native dimeric and inhibited dimeric pUL26N were deposited with the Protein Data Bank under entry codes 4v0t, 4v07, and 4v08, respectively. A preliminary dataset of the monomeric pUL26N at 2.5 Å resolution was deposited with entry code 4cx8.

### Small-angle X-ray scattering

SAXS data were recorded at beamline P12 of the EMBL outstation at PETRA III, DESY, Hamburg [90], using a PILATUS 2M pixel detector, a sample-to-detector distance of 3.1 m and a wavelength of 1.24 Å. Solutions contained 0.5 M NaCl, 50 mM Tris pH 7.5, 0.25 M imidazole, 5% glycerol, 50 mM urea and pUL26N as indicated. In all experiments the sample temperature was 283 K. Measurements covered the momentum transfer range 0.008 < *s* < 0.47 Å^-1^ (*s* = 4π sin(θ) / λ, where 2θ is the scattering angle and λ is the X-ray wavelength). To monitor radiation damage, 20 successive 50 ms exposures of protein solutions were compared, revealing no significant change. The data were normalized to the intensity of the transmitted beam and radially averaged. Scattering of the buffer was subtracted and the difference curves were scaled to unity protein concentration (1 mg/ml). For further data analysis, version 2.6.0 of the *ATSAS* package was used [91]. Form factors were generated from the monomeric and dimeric crystallographic models by means of the program FFMAKER. For subsequent curve fitting, the program OLIGOMER [63] was used. An *ab initio* model corresponding to the highest protein concentration in the presence of MgCl_2_ was generated using the programs DAMMIF [92], DAMAVER [93] and DAMMIN [64] *via* the PRIMUS interface [63], in "slow" mode and without imposing particle symmetry.

The scattering data, structural models and curve fittings of dimeric PrV pUL26N were deposited with the small-angle scattering biological data bank (SASBDB) with entry code SASDA58 [94].

### Accession numbers/ID numbers of genes and proteins

PrV UL26: Gene ID 2952508

HSV-1 UL26: Gene ID 2703453

PrV UL26.5: Gene ID 2952525

HSV-1 UL26.5: Gene ID 2703454

PrV pUL26: Q83417 in UniProtKB, S21.001 in MEROPS [95]

PrV pUL26N: Amino-acid residues 1–224 of Q83417 in UniProtKB, S21.001 in MEROPS [95]

PrV pUL26C: Amino-acid residues 226–524 of Q83417 in UniProtKB

PrV pUL26.5: Q83418 in UniProtKB

KA: Q2HRB6 in UniProtKB, S21.006 in MEROPS [95]

## Supporting Information

S1 Fig Superposition and B-factor coloring of dimeric PrV assemblin with and without bound inhibitor. Comparison of dimeric and inhibited dimeric pUL26N from PrV. B-factors less than 30 Å^2^ are colored deep blue. B-factors above 80 Å^2^ are colored red. High B-factors are observed primarily for some loops and at the far side of the dimer. The dimer interface helices, as well as the oxyanion-hole loops are well ordered according to low B-factors.
(TIF)

S2 Fig Superposition of representative assemblin structures from all herpesvirus subfamilies. Orthogonal views (A, B) of superposed dimeric assemblin structures from the alphaherpesviruses pseudorabies virus (pdb entry 4v08, this report, colored red) and varicella zoster virus (pdb entry 1vzv, colored orange), the betaherpesvirus human cytomegalovirus (pdb entry 1cmv, colored dark blue), and the gammaherpesvirus Kaposi's sarcoma-associated herpesvirus (pdb entry 1fl1, colored light blue). The black dashed line indicates the two-fold axis of the dimers.
(TIF)

S3 Fig Amino-acid sequence alignment of assemblins from different *herpesviridae* subfamilies. Alignment of amino-acid sequences of assemblins from different herpesvirus subfamilies with available structure models. Shown are the assemblins from the alphaherpesviruses: pseudorabies virus (PrV, UniProtKB accession number Q83417), varicella zoster virus (VZV, UniProtKB accession number P09286) and human herpes simplex virus type 1 and 2 (HSV-1 and 2, UniProtKB accession numbers P10210 and Q69527, respectively) as well as the assemblins from the betaherpesvirus human cytomegalovirus (HCMV, UniProtKB accession number P16753) and the gammaherpesviruses Epstein-Barr virus (EBV, UniProtKB accession number P03234) and Kaposi's sarcoma-associated herpesvirus (KSHV, UniProtKB accession number O36607). Alignment was performed using the Clustal Omega Webservice on the EMBL-EBI website (http://www.ebi.ac.uk/Tools/msa/clustalo/) [39,40]. Annotation of the alignment was carried out using Aline [96]. Arrows and cylinders above the alignment correspond to β-strands and α-helices of dimeric PrV assemblin, respectively. The catalytic triad is labeled red; the oxyanion-hole loop is marked by blue background with the two consecutive, conserved arginine residues colored green and residues involved in the conserved hydrophobic interactions in the monomer or dimer of PrV pUL26N are labeled yellow. Asterisks mark residues that are identical in all aligned sequences, whereas dots and colons mark similar and highly similar residues, respectively. Five out of the ten residues of the oxyanion-hole loop are strictly conserved throughout assemblins.
(TIF)

S4 Fig Extended hydrogen-bond network of the oxyanion-hole and active site of dimeric PrV assemblin. Stereoview of the active site and oxyanion-hole loop (OHL) of inhibited dimeric pUL26N from PrV with the detailed hydrogen bond network. The OHL is shown in orange, the covalent bound diisopropyl phosphate in red and the catalytic triad in purple. Water molecules are shown as red spheres. All dashed lines illustrate hydrogen bonds in the range of 2.7 to 3.4 Å. The strictly conserved Arg136 forms the oxyanion hole with its peptide backbone N-H. Inhibitor binding mimics the transition state of the natural substrate.
(TIF)

S5 Fig SAXS analysis of pUL26N from PrV at various concentrations and in the presence or absence of MgCl_2_. (A) Concentration-normalized scattering from 5 mg/ml pUL26N in the absence of MgCl_2_ (black dots). The momentum transfer *s* is defined as 4π sin(θ) / λ, where 2θ is the scattering angle and λ = 1.24 Å is the X-ray wavelength. The fitted curve (corresponding to 50% monomer volume percentage, χ^2^ = 1.25) calculated using OLIGOMER [63] is shown in red. (B) Monomer volume fractions as determined by OLIGOMER [63], plotted against protein concentration. The estimated dissociation constants are based on the fitted curves. Black: buffer without MgCl_2_. Red: buffer containing 0.2 M MgCl_2_. An arrow indicates the approximate initial protein concentration in our crystallization drops. (C, D) Orthogonal views of the *ab initio* model calculated for pUL26N at 10 mg/ml in MgCl_2_-containing buffer. The final beads model from DAMMIN [64] was superposed onto the crystallographic model for the PrV pUL26N dimer (ribbon representation with different colors indicating subunits) by means of the program SUPCOMB [97].
(TIF)

S6 Fig C-terminally truncated KSHV assemblin (KA) in complex with helical-peptide mimetics (HPMs) differs from its native dimer. Superposition of A-chains of dimeric KA (pdb entry 2pbk, chain A is shown in light red, chain B is shown in red) with C-terminally truncated KA with bound helical-peptide mimetics (pdb entry 4p2t, chain A is shown in light blue, chain B of the dimer forming symmetry mate is shown in dark blue, molecules of the helical-peptide mimetics (HPMs) are shown in pink). The dashed green line indicates the approximate 80° rotation axis relating the monomers in the native and artificial dimer. The molecules of the HPMs imitate the truncated dimerization helix causing an artificial inactive dimer. This positions the C-termini close to each other in the truncated form (S7 Fig). Thus, any additional C-terminal residues of the full-length assemblin in complex with HPMs will prevent this dimerization mode by sterical hindrance. Helices corresponding to α7 and α8 in PrV assemblin are truncated in HPM complexes of KA (pdb entry 4p2t). These helices are colored dark gray in the model of full-length KA (pdb entry 2pbk). Loop regions are omitted for clarity and regions that differ from each other are also omitted in B-chains.
(TIF)

S7 Fig Artificial dimer of C-terminally truncated KSHV assemblin in complex with helical-peptide mimetics. Chain A and B of the artificial dimer of C-terminally truncated KA (pdb entry 4p2t) are shown in dark blue and light blue, respectively. The truncated major interface helices are substituted by three molecules of a helical-peptide mimetic (shown in pink). The truncated C-termini (labeled green) are in close proximity to each other. This assembly is hardly conceivable in full-length KA dimer, because the extended C-termini will cause sterical hindrance.
(TIF)

S8 Fig Monomeric PrV assemblin compared to truncated KSHV assemblin in complex with helical-peptide mimetics. Superposition of A-chains of monomeric pUL26N from PrV (shown in orange) and the truncated KA (pdb entry 4p2t, shown in blue) with helical-peptide mimetics (shown in pink). The oxyanion-hole loop (OHL) of aligned chain B of monomeric pUL26N from PrV is shown in black. Dashed lines represent parts of the polypeptide, which are not modeled. The OHL of dimeric pUL26N from PrV is additionally represented for comparison (shown in green).
(TIF)

S9 Fig Comparison of the loop connection β1-α2 from all available assemblin models. The loop connection β1-α2 in truncated KSHV assemblin in complex with HPMs differs from all assemblin structures. Conformational changes in truncated KA apparently lead to formation of an extended β-sheet with a symmetry mate, because of conformational changes of the loop β1-α2. In truncated KA, this loop is different to all other assemblin structures. Shown are orthogonal views (A, B) of a superposition of all assemblin structures known to date. The dimeric structures of PrV assemblin are colored red, the monomeric structure of PrV assemblin is colored orange and truncated KAs in complex with helical-peptide mimetics are colored blue. The pdb entries of shown structural models are 1at3, 1cmv, 1fl1, 1id4, 1iec, 1ied, 1ief, 1ieg, 1jq6, 1jq7, 1lay, 1njt, 1nju, 1nkk, 1nkm, 1o6e, 1vzv, 1wpo, 2pbk, 2wpo, 3njq, 4p2t, 4p3h, 4v0t, 4v07, and 4v08. The β5-β6 loops are omitted for clarity.
(TIF)

S10 Fig Helical-peptide mimetic binding-site of truncated KSHV assemblin with superposed monomeric PrV assemblin. Superposition of monomeric pUL26N from PrV (shown in orange) and truncated KA (pdb entry 4p2t, shown in blue) with helical-peptide mimetics (HPM, shown in pink). A detailed view onto the interface area is shown. Hydrophobic side chains involved in HPM binding are shown as well as potential HPM-binding side chains from PrV assemblin. The “hot-spot” residues [65] of KA are labeled. The corresponding residues of pUL26N from PrV assemblin are labeled in braces.
(TIF)

S11 Fig Binding site of the putative Mg^2+^ ion. The putative Mg^2+^ ion in the crystal structures of the dimer (shown in purple) and the inhibited dimer (shown in green) of pUL26N from PrV. Putative metal-binding sites are shown as big spheres and water molecules as smaller spheres. Numbers are distances in Å. Distances and bond angles strongly suggest Mg^2+^ ions with reasonable coordination sphere. The cations are coordinated by water molecules. One of them connects two aspartate side chains of different dimers by hydrogen bonds.
(TIF)

S1 Table Overview of SAXS experiments and curve fitting results. Listed are the concentrations of pUL26N and MgCl_2_, the monomer volume fractions determined by means of the program OLIGOMER and the corresponding χ^2^ values from the curve fitting procedure.
(DOCX)

S1 Text Detailed comparison of monomeric pUL26N and HPM complexes of truncated KSHV assemblin. (DOCX)
