# Correction: Dimerization-Induced Allosteric Changes of the Oxyanion-Hole Loop Activate the Pseudorabies Virus Assemblin pUL26N, a Herpesvirus Serine Protease

**DOI:** 10.1371/journal.ppat.1005506

**Published:** 2016-03-11

**Authors:** Martin Zühlsdorf, Sebastiaan Werten, Barbara G. Klupp, Gottfried J. Palm, Thomas C. Mettenleiter, Winfried Hinrichs

There is an error in the final sentence of the paragraph titled “Monomer-dimer equilibrium” in the Results section. The correct sentence is: The dissociation constants with and without MgCl2 can be estimated to 50μM and 200μM, respectively (S5 Fig).
