# Supplementary figures and images for: Dimerization-Induced Allosteric Changes of the Oxyanion-Hole Loop Activate the Pseudorabies Virus Assemblin pUL26N, a Herpesvirus Serine Protease
Source: PLoS Pathog. 2015 Jul 10;11(7):e1005045. doi: 10.1371/journal.ppat.1005045 (PMC4498786; doi:10.1371/journal.ppat.1005045)

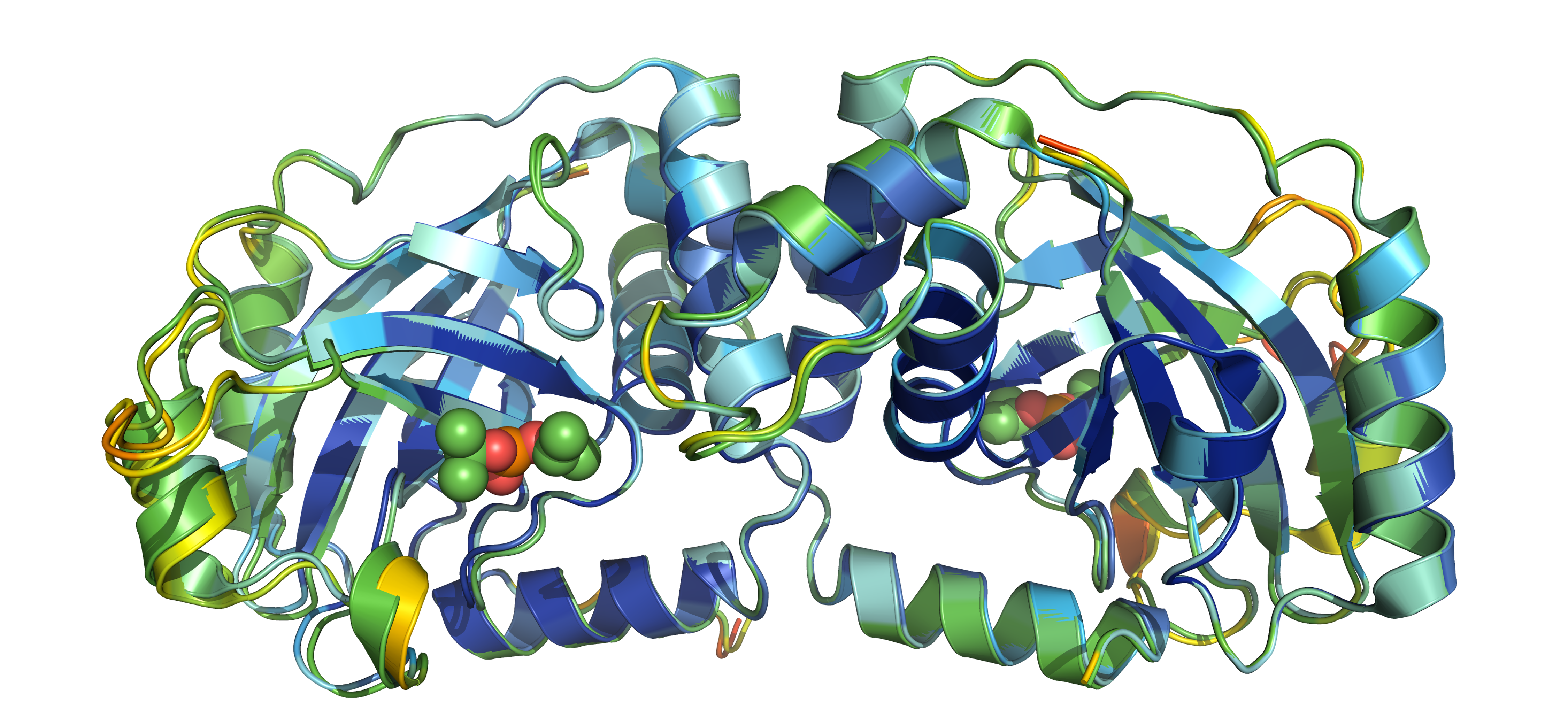

Supplement: S1 Fig — Comparison of dimeric and inhibited dimeric pUL26N from PrV. B-factors less than 30 Å2 are colored deep blue. B-factors above 80 Å2 are colored red. High B-factors are observed primarily for some loops and at the far side of the dimer. The dimer interface helices, as well as the oxyanion-hole loops are well ordered according to low B-factors. (TIF) [file ppat.1005045.s001.tif]

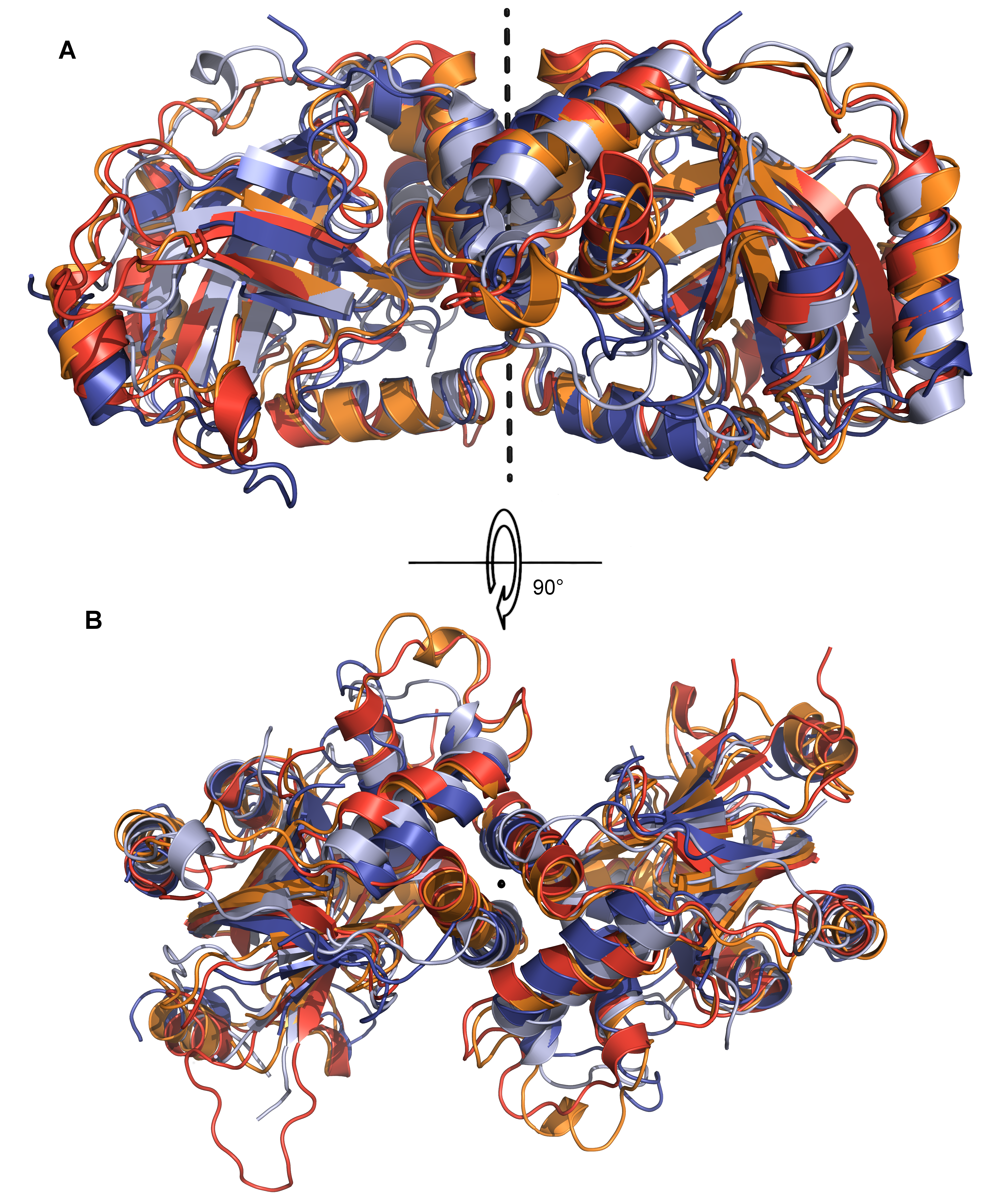

Supplement: S2 Fig — Orthogonal views (A, B) of superposed dimeric assemblin structures from the alphaherpesviruses pseudorabies virus (pdb entry 4v08, this report, colored red) and varicella zoster virus (pdb entry 1vzv, colored orange), the betaherpesvirus human cytomegalovirus (pdb entry 1cmv, colored dark blue), and the gammaherpesvirus Kaposi's sarcoma-associated herpesvirus (pdb entry 1fl1, colored light blue). The black dashed line indicates the two-fold axis of the dimers. (TIF) [file ppat.1005045.s002.tif]

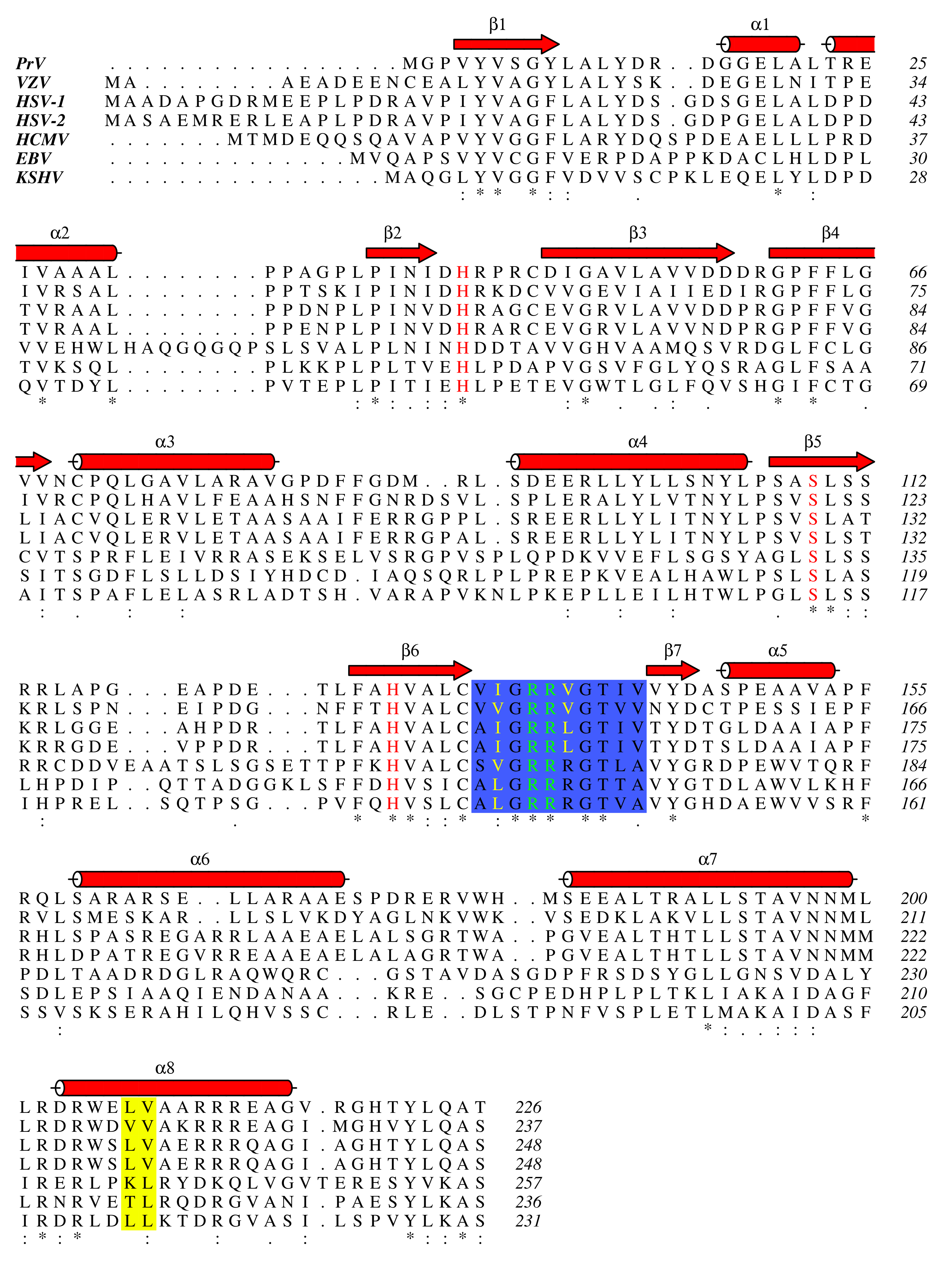

Supplement: S3 Fig — Alignment of amino-acid sequences of assemblins from different herpesvirus subfamilies with available structure models. Shown are the assemblins from the alphaherpesviruses: pseudorabies virus (PrV, UniProtKB accession number Q83417), varicella zoster virus (VZV, UniProtKB accession number P09286) and human herpes simplex virus type 1 and 2 (HSV-1 and 2, UniProtKB accession numbers P10210 and Q69527, respectively) as well as the assemblins from the betaherpesvirus human cytomegalovirus (HCMV, UniProtKB accession number P16753) and the gammaherpesviruses Epstein-Barr virus (EBV, UniProtKB accession number P03234) and Kaposi's sarcoma-associated herpesvirus (KSHV, UniProtKB accession number O36607). Alignment was performed using the Clustal Omega Webservice on the EMBL-EBI website (http://www.ebi.ac.uk/Tools/msa/clustalo/) [39,40]. Annotation of the alignment was carried out using Aline [96]. Arrows and cylinders above the alignment correspond to β-strands and α-helices of dimeric PrV assemblin, respectively. The catalytic triad is labeled red; the oxyanion-hole loop is marked by blue background with the two consecutive, conserved arginine residues colored green and residues involved in the conserved hydrophobic interactions in the monomer or dimer of PrV pUL26N are labeled yellow. Asterisks mark residues that are identical in all aligned sequences, whereas dots and colons mark similar and highly similar residues, respectively. Five out of the ten residues of the oxyanion-hole loop are strictly conserved throughout assemblins. (TIF) [file ppat.1005045.s003.tif]

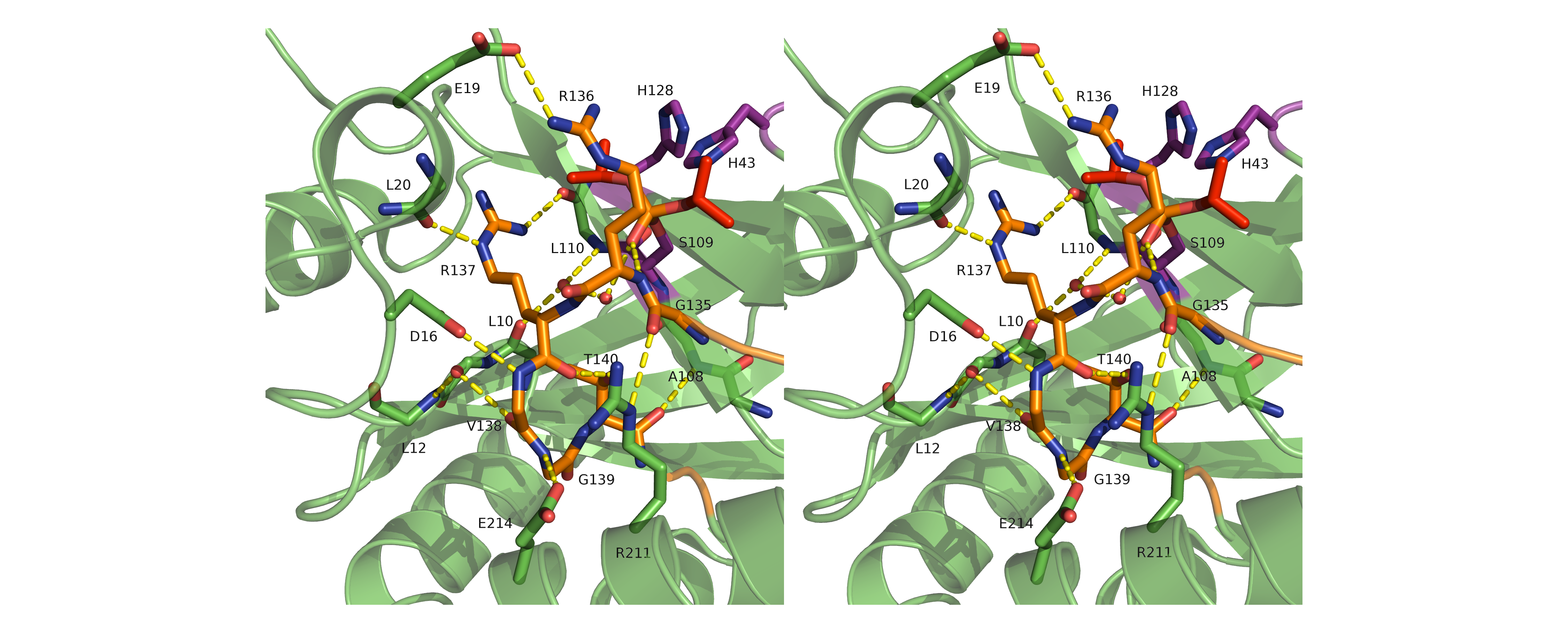

Supplement: S4 Fig — Stereoview of the active site and oxyanion-hole loop (OHL) of inhibited dimeric pUL26N from PrV with the detailed hydrogen bond network. The OHL is shown in orange, the covalent bound diisopropyl phosphate in red and the catalytic triad in purple. Water molecules are shown as red spheres. All dashed lines illustrate hydrogen bonds in the range of 2.7 to 3.4 Å. The strictly conserved Arg136 forms the oxyanion hole with its peptide backbone N-H. Inhibitor binding mimics the transition state of the natural substrate. (TIF) [file ppat.1005045.s004.tif]

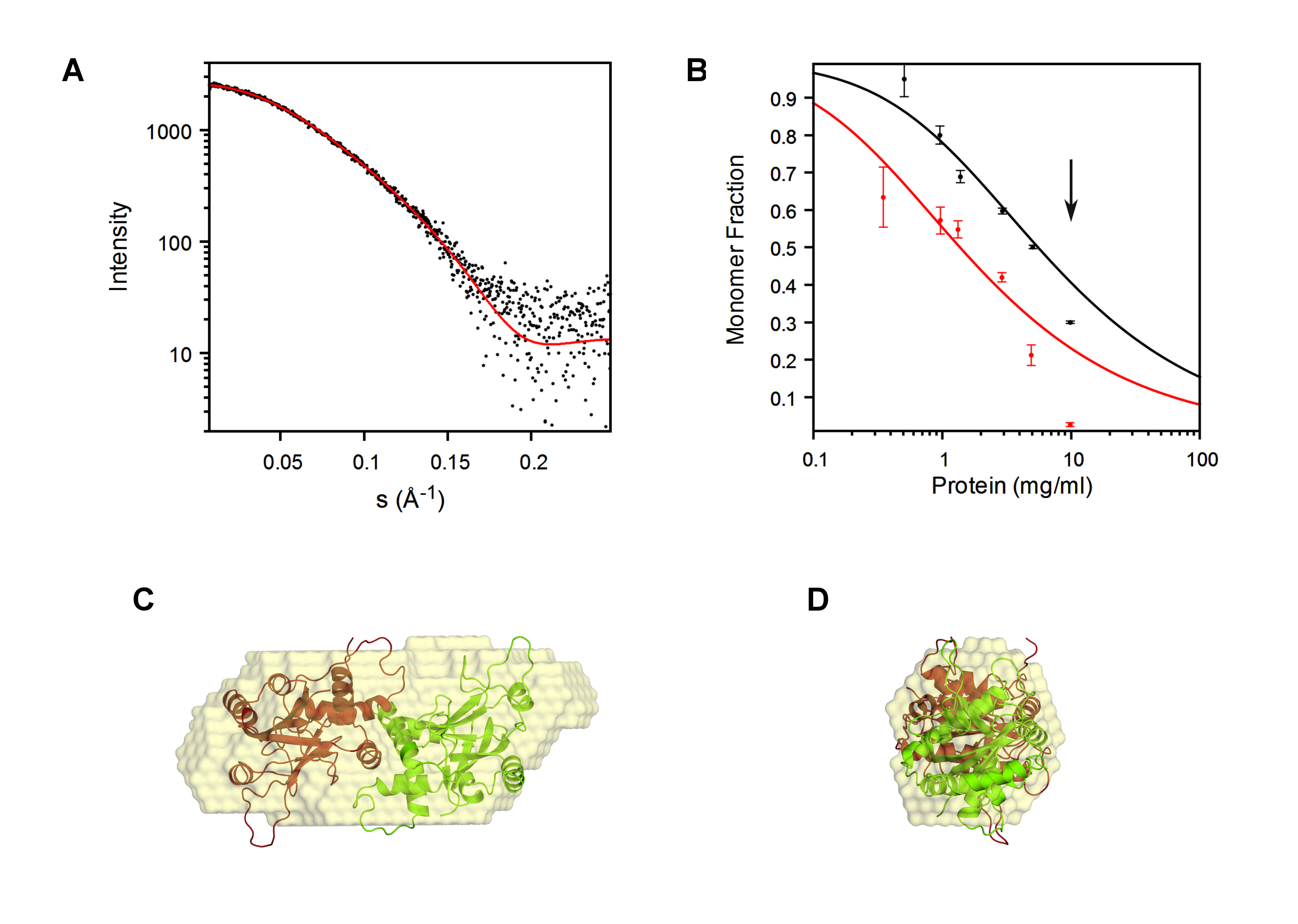

Supplement: S5 Fig — (A) Concentration-normalized scattering from 5 mg/ml pUL26N in the absence of MgCl2 (black dots). The momentum transfer s is defined as 4π sin(θ) / λ, where 2θ is the scattering angle and λ = 1.24 Å is the X-ray wavelength. The fitted curve (corresponding to 50% monomer volume percentage, χ2 = 1.25) calculated using OLIGOMER [63] is shown in red. (B) Monomer volume fractions as determined by OLIGOMER [63], plotted against protein concentration. The estimated dissociation constants are based on the fitted curves. Black: buffer without MgCl2. Red: buffer containing 0.2 M MgCl2. An arrow indicates the approximate initial protein concentration in our crystallization drops. (C, D) Orthogonal views of the ab initio model calculated for pUL26N at 10 mg/ml in MgCl2-containing buffer. The final beads model from DAMMIN [64] was superposed onto the crystallographic model for the PrV pUL26N dimer (ribbon representation with different colors indicating subunits) by means of the program SUPCOMB [97]. (TIF) [file ppat.1005045.s005.tif]

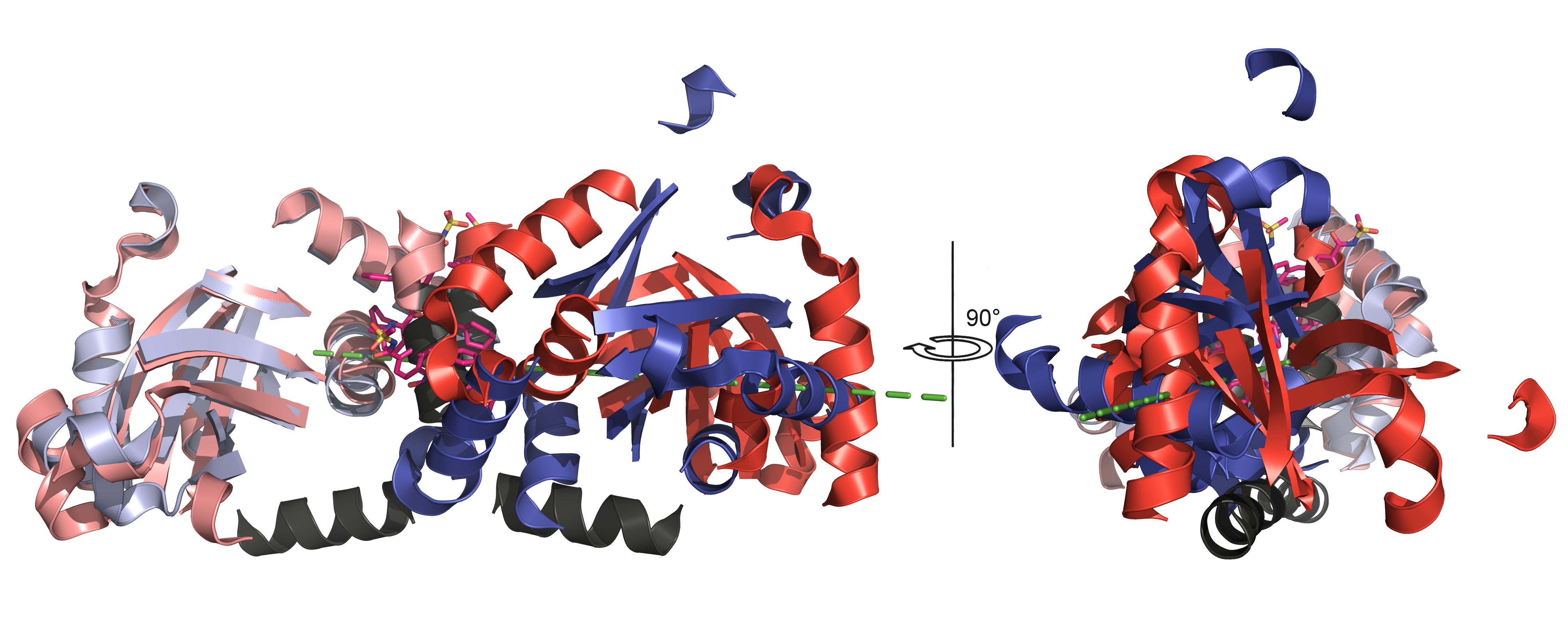

Supplement: S6 Fig — Superposition of A-chains of dimeric KA (pdb entry 2pbk, chain A is shown in light red, chain B is shown in red) with C-terminally truncated KA with bound helical-peptide mimetics (pdb entry 4p2t, chain A is shown in light blue, chain B of the dimer forming symmetry mate is shown in dark blue, molecules of the helical-peptide mimetics (HPMs) are shown in pink). The dashed green line indicates the approximate 80° rotation axis relating the monomers in the native and artificial dimer. The molecules of the HPMs imitate the truncated dimerization helix causing an artificial inactive dimer. This positions the C-termini close to each other in the truncated form (S7 Fig). Thus, any additional C-terminal residues of the full-length assemblin in complex with HPMs will prevent this dimerization mode by sterical hindrance. Helices corresponding to α7 and α8 in PrV assemblin are truncated in HPM complexes of KA (pdb entry 4p2t). These helices are colored dark gray in the model of full-length KA (pdb entry 2pbk). Loop regions are omitted for clarity and regions that differ from each other are also omitted in B-chains. (TIF) [file ppat.1005045.s006.tif]

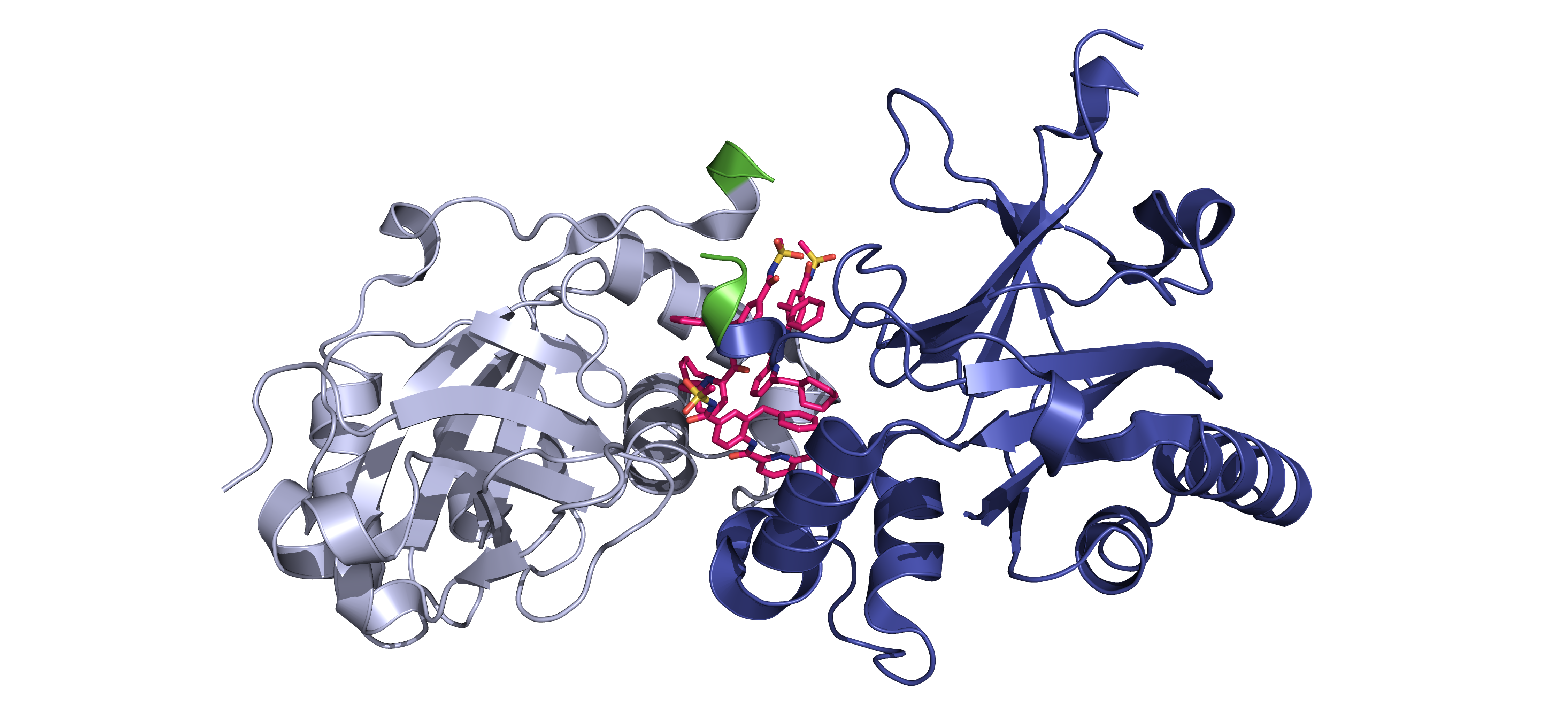

Supplement: S7 Fig — Chain A and B of the artificial dimer of C-terminally truncated KA (pdb entry 4p2t) are shown in dark blue and light blue, respectively. The truncated major interface helices are substituted by three molecules of a helical-peptide mimetic (shown in pink). The truncated C-termini (labeled green) are in close proximity to each other. This assembly is hardly conceivable in full-length KA dimer, because the extended C-termini will cause sterical hindrance. (TIF) [file ppat.1005045.s007.tif]

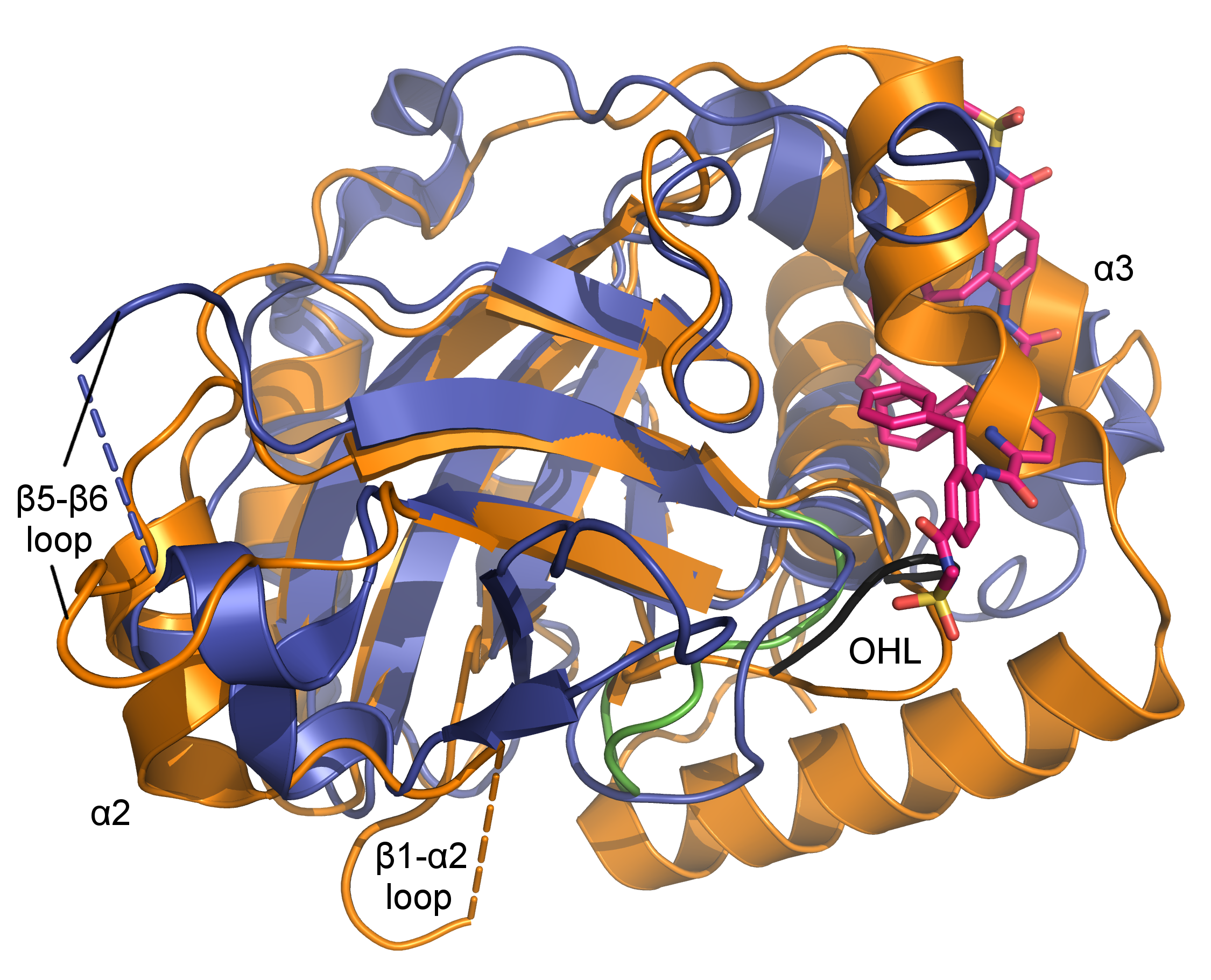

Supplement: S8 Fig — Superposition of A-chains of monomeric pUL26N from PrV (shown in orange) and the truncated KA (pdb entry 4p2t, shown in blue) with helical-peptide mimetics (shown in pink). The oxyanion-hole loop (OHL) of aligned chain B of monomeric pUL26N from PrV is shown in black. Dashed lines represent parts of the polypeptide, which are not modeled. The OHL of dimeric pUL26N from PrV is additionally represented for comparison (shown in green). (TIF) [file ppat.1005045.s008.tif]

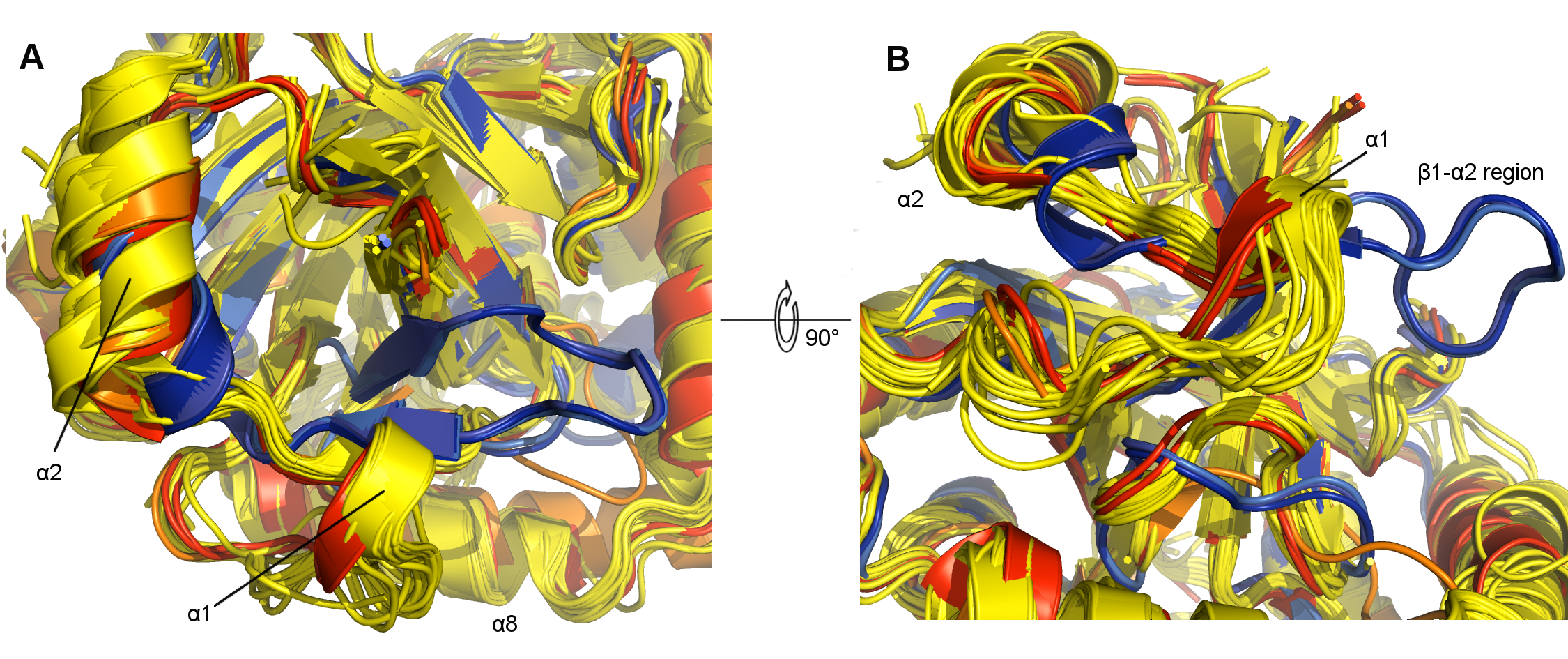

Supplement: S9 Fig — The loop connection β1-α2 in truncated KSHV assemblin in complex with HPMs differs from all assemblin structures. Conformational changes in truncated KA apparently lead to formation of an extended β-sheet with a symmetry mate, because of conformational changes of the loop β1-α2. In truncated KA, this loop is different to all other assemblin structures. Shown are orthogonal views (A, B) of a superposition of all assemblin structures known to date. The dimeric structures of PrV assemblin are colored red, the monomeric structure of PrV assemblin is colored orange and truncated KAs in complex with helical-peptide mimetics are colored blue. The pdb entries of shown structural models are 1at3, 1cmv, 1fl1, 1id4, 1iec, 1ied, 1ief, 1ieg, 1jq6, 1jq7, 1lay, 1njt, 1nju, 1nkk, 1nkm, 1o6e, 1vzv, 1wpo, 2pbk, 2wpo, 3njq, 4p2t, 4p3h, 4v0t, 4v07, and 4v08. The β5-β6 loops are omitted for clarity. (TIF) [file ppat.1005045.s009.tif]

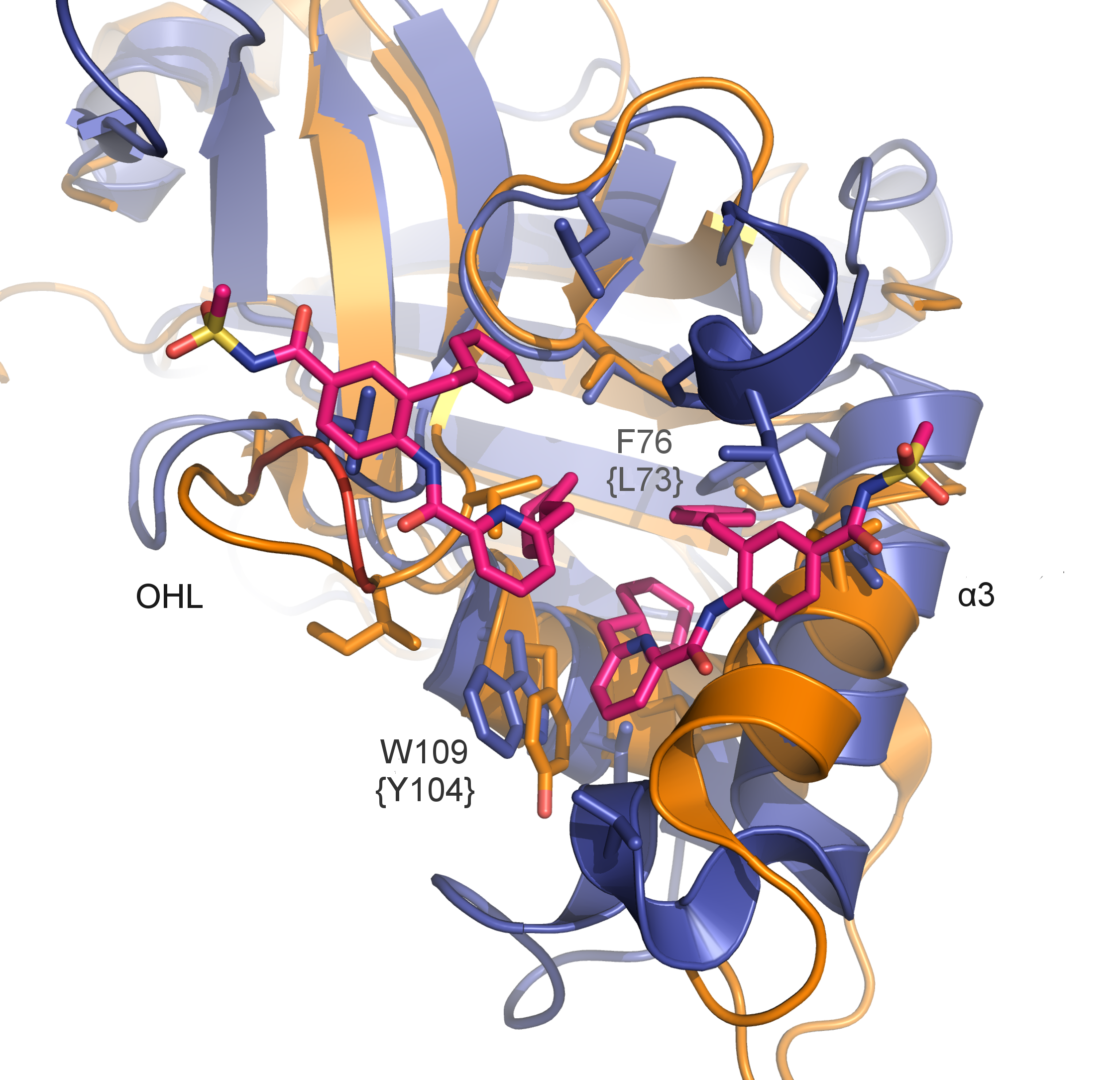

Supplement: S10 Fig — Superposition of monomeric pUL26N from PrV (shown in orange) and truncated KA (pdb entry 4p2t, shown in blue) with helical-peptide mimetics (HPM, shown in pink). A detailed view onto the interface area is shown. Hydrophobic side chains involved in HPM binding are shown as well as potential HPM-binding side chains from PrV assemblin. The “hot-spot” residues [65] of KA are labeled. The corresponding residues of pUL26N from PrV assemblin are labeled in braces. (TIF) [file ppat.1005045.s010.tif]

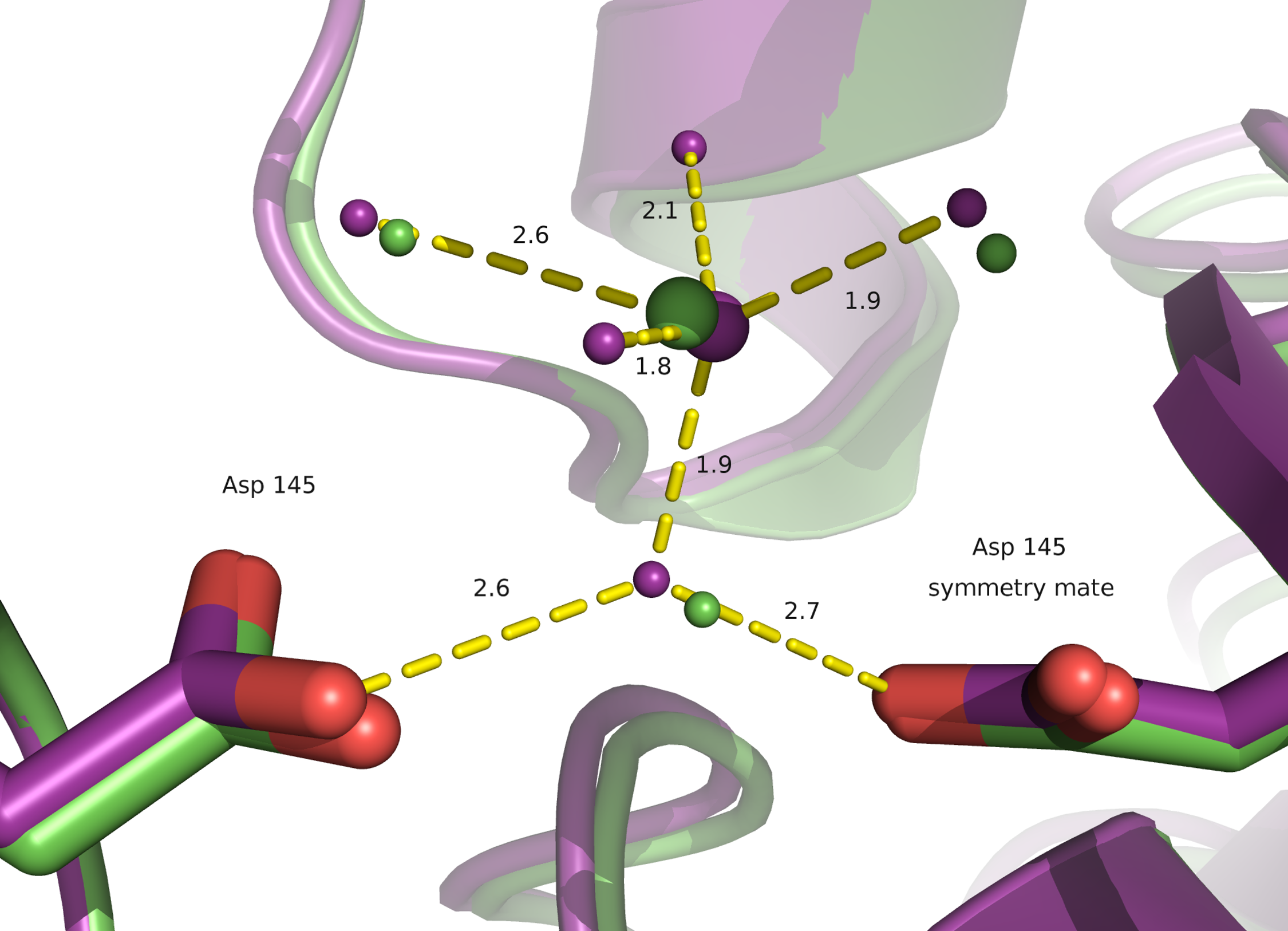

Supplement: S11 Fig — The putative Mg2+ ion in the crystal structures of the dimer (shown in purple) and the inhibited dimer (shown in green) of pUL26N from PrV. Putative metal-binding sites are shown as big spheres and water molecules as smaller spheres. Numbers are distances in Å. Distances and bond angles strongly suggest Mg2+ ions with reasonable coordination sphere. The cations are coordinated by water molecules. One of them connects two aspartate side chains of different dimers by hydrogen bonds. (TIF) [file ppat.1005045.s011.tif]
