# Supplementary material for: Dimerization-Induced Allosteric Changes of the Oxyanion-Hole Loop Activate the Pseudorabies Virus Assemblin pUL26N, a Herpesvirus Serine Protease
Source: PLoS Pathog. 2015 Jul 10;11(7):e1005045. doi: 10.1371/journal.ppat.1005045 (PMC4498786; doi:10.1371/journal.ppat.1005045)
Supplement: S1 Table — Listed are the concentrations of pUL26N and MgCl2, the monomer volume fractions determined by means of the program OLIGOMER and the corresponding χ2 values from the curve fitting procedure. (DOCX) [file ppat.1005045.s012.docx]

| **pUL26N (mg/ml)** | **MgCl_2_** | **Monomer fraction** | **χ^2^** |
| --- | --- | --- | --- |
| 0.51 | – | 0.95 ± 0.05 | 1.04 |
| 0.96 | – | 0.80 ± 0.02 | 1.10 |
| 1.39 | – | 0.69 ± 0.02 | 0.99 |
| 2.94 | – | 0.60 ± 0.01 | 1.05 |
| 5.06 | – | 0.50 ± 0.01 | 1.25 |
| 9.89 | – | 0.300 ± 0.003 | 1.54 |
| 0.35 | 0.2 M | 0.63 ± 0.08 | 1.04 |
| 0.97 | 0.2 M | 0.57 ± 0.04 | 1.01 |
| 1.33 | 0.2 M | 0.55 ± 0.02 | 0.99 |
| 2.91 | 0.2 M | 0.42 ± 0.01 | 1.02 |
| 4.91 | 0.2 M | 0.21 ± 0.03 | 0.99 |
| 9.83 | 0.2 M | 0.03 ± 0.01 | 1.36 |
