# Supplementary material for: Dimerization-Induced Allosteric Changes of the Oxyanion-Hole Loop Activate the Pseudorabies Virus Assemblin pUL26N, a Herpesvirus Serine Protease
Source: PLoS Pathog. 2015 Jul 10;11(7):e1005045. doi: 10.1371/journal.ppat.1005045 (PMC4498786; doi:10.1371/journal.ppat.1005045)
Supplement: S1 Text — (DOCX) [file ppat.1005045.s013.docx]

The core of the truncated KA in complex with HPMs is similar to the monomeric and dimeric structures of PrV assemblin (S8 Fig). There are, however, differences at the dimer interface, the OHL, the β1-α2 loop including helix α2, and the β5-β6 loop. These differences originate most likely from the truncation of helices α7 and α8, because the altered interface affects the OHL and induces formation of two β-strands instead of α1. These strands form an extended β-sheet, which results in a conformation of the loop connection of strand β1 and helix α2 being different to all other structures of assemblins (S9 Fig). In monomeric PrV assemblin, the changes in this area are much smaller, because helices α7 and α8 are not truncated. In dimeric full-length KA without bound inhibitors (pdb entry 1fl1), the loop β1-α2 is missing in the model due to weak electron density. The loop becomes ordered and forms α1 upon substrate binding as seen in a model with a bound substrate analog (pdb entry 2pbk).

However, the OHL conformation of the HPM complexes is different from monomeric as well as dimeric PrV assemblin. Most probably, the conformation of this loop in the HPM complexes is solely an artifact of packing contacts. The structures of truncated KA with HPMs already revealed a displacement of the OHL and loss of the oxyanion hole, which drastically decreases the activity. In monomeric PrV assemblin, it is much more in contact with the dimerization area. This is possible because of the helices α7 and α8, which are missing completely in HPM complexes of KA due to truncation.

The alternative hydrophobic interaction of the OHL in monomeric (Val138) and dimeric (Ile134) PrV assemblin with helix α8 that we have described in this report does not have an equivalent in the HPM complex. This is a direct consequence of the truncation of the C-terminal helices in KA, since these contribute the hydrophobic side chains (Leu212 and Leu213 in KA, Leu207 and Val208 in PrV, respectively) that tether the aliphatic residues. According to our sequence alignment (S3 Fig), Val138 of PrV assemblin corresponds to arginine residues in beta- and gammaherpesvirus assemblins. Given the length and physicochemical properties of the arginine side-chain, a positioning of the OHL similar to that of monomeric PrV assemblin in the presence of the C-terminal helix is unlikely for beta- and gammaherpesvirus assemblins. This may explain the reported disorder of the C-terminal helices of monomeric full-length KA in solution [28].

In truncated KA, binding of HPMs is established by Trp109 (α4) [43] and maybe Phe76 (α3), the so-called “hot spots” [65], among several additional hydrophobic residues (S10 Fig). The hot spots Trp109 and Phe76 correspond to Tyr (α4) and Leu (α3), respectively, in PrV, VZV, HSV-1 and HSV-2 assemblins (S3 Fig). Neither Leu nor Tyr are considered as hot spot residues [65] and thus, binding of these HPMs is likely weakened. Additionally, in alphaherpesvirus assemblins the HPMs have to compete against the likely ordered C-terminal helices for binding, further weakening the association. Screening for suitable mimetics is necessary to achieve specific and efficient inactivation of alphaherpesvirus proteases.
